# Behavioral and Molecular Effects Induced by Cannabidiol and Valproate Administration in the GASH/Sal Model of Acute Audiogenic Seizures

**DOI:** 10.3389/fnbeh.2020.612624

**Published:** 2021-01-22

**Authors:** Giselda Cabral-Pereira, David Sánchez-Benito, Sandra M. Díaz-Rodríguez, Jaime Gonçalves, Consuelo Sancho, Orlando Castellano, Luis J. Muñoz, Dolores E. López, Ricardo Gómez-Nieto

**Affiliations:** ^1^Institute of Neuroscience of Castilla y León (INCYL), University of Salamanca, Salamanca, Spain; ^2^Institute for Biomedical Research of Salamanca (IBSAL), University of Salamanca, Salamanca, Spain; ^3^Department of Cell Biology and Pathology, Faculty of Medicine, University of Salamanca, Salamanca, Spain; ^4^Department of Physiology and Pharmacology, Faculty of Medicine, University of Salamanca, Salamanca, Spain; ^5^Animal Research and Service Center, University of Salamanca, Salamanca, Spain

**Keywords:** animal models, antiepileptic drugs, cannabis, epilepsy, gene expression, inferior colliculus (IC), valproic acid, drug interactions

## Abstract

Despite evidence that supports cannabidiol (CBD) as an anticonvulsant agent, there remains controversy over the antiseizure efficacy, possible adverse effects, and synergistic interactions with classic antiepileptics such as valproate (VPA). The genetic audiogenic seizure hamster from the University of Salamanca (GASH/Sal) is a reliable experimental model of generalized tonic–clonic seizures in response to intense sound stimulation. The present study examines the behavioral and molecular effects of acute and chronic intraperitoneal administrations of VPA (300 mg/kg) and CBD (100 mg/kg) on the GASH/Sal audiogenic seizures, as well as the coadministration of both drugs. The GASH/Sal animals were examined prior to and after the corresponding treatment at 45 min, 7 days, and 14 days for seizure severity and neuroethology, open-field behaviors, body weight variations, and various hematological and biochemical parameters. Furthermore, the brain tissue containing the inferior colliculus (so-called epileptogenic nucleus) was processed for reverse transcription–quantitative polymerase chain reaction analysis to determine the treatment effects on the gene expression of neuronal receptors associated with drug actions and ictogenesis. Our results indicated that single dose of VPA helps prevent the animals from getting convulsions, showing complete elimination of seizures, whereas 7 days of chronic VPA treatment had few effects in seizure behaviors. Acute CBD administration showed subtle attenuation of seizure behaviors, increasing seizure latency and decreasing the duration of the convulsion phase, but without entirely seizure abolition. Chronic CBD treatments had no significant effects on sound-induced seizures, although some animals slightly improved seizure severity. Acute and chronic CBD treatments have no significant adverse effects on body weight, hematological parameters, and liver function, although locomotor activity was reduced. The combination of VPA and CBD did not alter the therapeutic outcome of the VPA monotherapy, showing no apparent synergistic effects. As compared to sham animals, chronic treatments with CBD caused abnormal mRNA expression levels for *Trpv1, Adora1, Slc29a1*, and *Cnr1* genes, whereas no differences in gene expression were found for *Htr1a* and *Sigmar1*. Our study shed light on the behavioral and molecular effects of CBD and VPA on the GASH/Sal model and constituted the basis to develop further studies on the pharmacological effects of CBD and its interactions with other anticonvulsants.

## Introduction

Epilepsy syndromes are considered neurological disorders characterized by an enduring predisposition to generate epileptic seizures that can be accompanied by a wide spectrum of behavioral and neuropsychiatric conditions (Kanner et al., 2012; Fisher et al., 2014). Among the myriad of epilepsy symptoms, seizures are one of the most important and notable signs that are associated with the abnormal excessive or synchronous neuronal activity in the brain (Fisher et al., 2005). Despite the numerous antiseizure drugs with different modes of action, a large number of patients confirm pharmacoresistance, that is, the failure to achieve seizure control with a trial of an anticonvulsant medication given at the appropriate dosage (Kwan et al., 2009; Sharma et al., 2015). Uncontrolled seizure is closely linked to an increased risk of pharmacoresistance that is the major responsible for boosting mortality, and no promising therapeutic treatments are currently available to prevent the emergence of pharmacoresistance (reviewed in Sharma et al., 2015). Thus, the high incidence of epilepsy (more than 60 million people worldwide) and the increasing number of antiseizure drug–resistant patients (~40% of the cases) call for the encouragement of evaluating new anticonvulsant compounds. Toward this end, experimental animal models of epilepsies and seizures have proven to be one of the biggest assets in the research programs that focused on the discovery and development of new antiseizure drugs (Nef, 2001; Löscher, 2011). In this context, cannabidiol (CBD), the major non-psychoactive phytocannabinoid present in the *Cannabis sativa* plant, has been increasingly attracting therapeutic interest, particularly because of its anticonvulsive actions and favorable side effect profile in preclinical models of seizures and epilepsy (Devinsky et al., 2014; Ibeas Bih et al., 2015). Among all of the animal models used to study the anticonvulsant properties of CBD, there is less evidence for CBD's effects on audiogenic seizure (AGS) models of genetic origin (reviewed in Lazarini-Lopes et al., 2020). AGSs are frequent in rodents that exhibited generalized clonic or tonic–clonic convulsive muscle contractions caused by excessive or abnormal neuronal firing in response to intense sound stimulation. It is well-established that acutely induced AGSs depend on brainstem substrates (Faingold, 1999; Garcia-Cairasco, 2002), whereas repetitions of AGS (after an audiogenic kindling protocol) further recruit limbic brain regions (Romcy-Pereira and Garcia-Cairasco, 2003; Dutra et al., 2009). In contrast to traditional models of epilepsy induced by chemical or electrical means, the genetically AGS rodents offer several advantages: (1) the AGS susceptibility is inherited and does not require any experimental procedure to become susceptible, thus avoiding incompatibilities with antiseizure drugs administration; (2) the innate occurring seizures can be elicited at will by an investigator, as the specific trigger is a sound; and (3) the substantial characterization of behavioral, cellular, and molecular alterations available that potentiates their usefulness in antiepileptic drug screening and elucidating mechanisms underlying ictogenesis (reviewed in Kandratavicius et al., 2014; Bosque et al., 2019). The genetic AGS hamster from Salamanca (GASH/Sal) exhibits susceptibility to sound-induced seizures and is the only susceptible hamster strain that is scientific research available (Muñoz et al., 2017). The GASH/Sal has recently gathered attention for the amount of interesting studies related to the neuroethological (Barrera-Bailón et al., 2013, 2017), electrophysiological (Carballosa-Gonzalez et al., 2013), neurochemical (Prieto-Martín et al., 2017), molecular (López-López et al., 2017; Díaz-Casado et al., 2020; Díaz-Rodríguez et al., 2020), and morphological (Sánchez-Benito et al., 2017, 2020) substrates underlying AGS. GASH/Sal animals, from 2 to 4 months of age at which susceptibility reached its maximum, undergoes generalized tonic–clonic seizures that are characterized by a short latency period after loud acoustic stimulation, followed by phases of wild running, convulsions, and stupor (Muñoz et al., 2017). Anticonvulsant drugs such as valproic acid (VPA), phenobarbital, and lamotrigine are highly effective in suppressing sound-induced seizures in the GASH/Sal (Barrera-Bailón et al., 2013, 2017), and hence GASH/Sal animals are suitable to examine the efficacy of antiepileptic agents (Werner and Coveñas, 2017). The overall goal of the present study was to make a preliminary determination of the potential anticonvulsant effects of CBD treatment in the GASH/Sal model following acute and chronic intraperitoneal administration, as well as the possible adverse effects on the hematologic profile, liver function, body weight, general locomotor activity levels, and emotionality. As no data exist regarding synergistic combinations of anticonvulsant agents with CBD in genetically AGS models, we also study the effects of concomitant treatment with VPA, an effective antiepileptic drug in the GASH/Sal model (Barrera-Bailón et al., 2013). Thus, by using a neuroethological approach (Garcia-Cairasco et al., 1996, 2004), we carried out a quantitative behavioral analysis of the AGS phases after the treatments with VPA, CBD, or the combination of both drugs. As occurred in other AGS rodent models, activation of the auditory pathway is required for seizure development in the GASH/Sal model (Garcia-Cairasco, 2002; Muñoz et al., 2017). In fact, the innate AGS susceptibility of the GASH/Sal model lies in an upward spread of abnormal glutamatergic transmission throughout the primary acoustic pathway to the inferior colliculus, a critical integration center in the auditory midbrain that is considered the epileptogenic region (Sánchez-Benito et al., 2020). Furthermore, recent studies have found disrupted gene expression profiles in the inferior colliculus of the GASH/Sal under free-seizure conditions and after sound-induced seizures that are associated with seizure susceptibility and altered regulation of neuronal excitability (López-López et al., 2017; Damasceno et al., 2020; Díaz-Casado et al., 2020; Díaz-Rodríguez et al., 2020). In view of this, it becomes of interest to compare patterns of gene expression after each of the treatments in the inferior colliculus of the GASH/Sal, particularly when CBD is coadministered with VPA. Among the large variety of protein channel and receptors targeted by the CBD (reviewed in Franco and Perucca, 2019), we selected those involved in the regulation of neuronal excitability and mechanism of CBD action. At the completion of each chronic drug treatment, we therefore determined the gene expression levels of the following neuronal receptors: the transient receptor potential of vanilloid type 1 (*Trpv1*), the 5-hydroxytryptamine (serotonin) receptor 1A (*5-Htr1a*), the sigma non-opioid intracellular receptor 1 (*Sigmar1*), the adenosine A1 receptor (*Adora1*), the equilibrative nucleoside transporter 1 (*Slc29a1*), and the cannabinoid receptor 1 (*Cnr1*). Overall, our results provide valuable information on the behavioral and molecular effects of VPA, CBD, and the combination of both drugs on the acute AGS of the GASH/Sal model. From a technical point of view, the present study is highly valuable as it combines a multitechnical approach to generate correlated results at behavioral and molecular levels that constituted the basis for further analysis of the CBD effects and its interactions with other anticonvulsant agents.

## Materials and Methods

### Experimental Animals and Ethical Statement

A total of 38 GASH/Sal animals from the inbred strain maintained at the vivarium of the University of Salamanca (USAL, Spain) were used in this study. All the GASH/Sal animals were males, 4 months of age, as it is stated that the GASH/Sal strain exhibits the maximum susceptibility to seizures from 2 to 4 months of age (Muñoz et al., 2017). All procedures and experimental protocols were performed in accordance with the guidelines of the European Communities Council Directive (2010/63/UE) for the care and use of laboratory animals and approved by the Bioethics Committee of the University of Salamanca (application no. 380). All efforts were made to minimize the number of animals and their suffering. The animals were maintained in Eurostandard Type III cages (Tecniplast, Italy), with Lignocel bedding (Rettenmaier Iberica), 14/10 light–dark cycle, and 22–24°C room temperature with *ad libitum* access to food (Tecklad Global 2918 irradiated diet) and water. Communities of three to four animals were housed in groups until the beginning of the study, when they were individually caged 24 h prior to drug administration. To avoid the influence of the circadian rhythm, each of the different tests was performed at the same time of day.

### Experimental Design and Drug Administration

**Figure 1:**
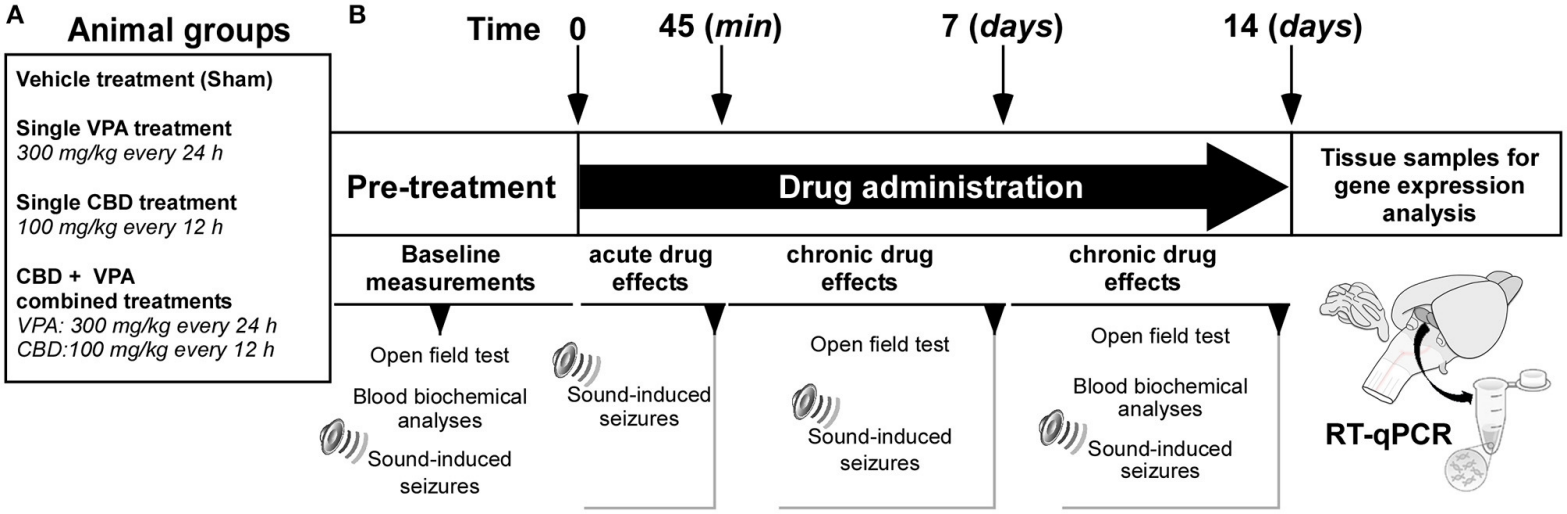
Experimental design and timeline. **(A)** Animal groups and drug treatments used in the study. **(B)** Experimental time schedules. Basal measurements were conducted prior to the initiation of the treatments. Acute effects of each drug treatment on sound-induced seizures were evaluated 45 min after the first intraperitoneal administration. Chronic effects of each drug treatment on sound-induced seizures were evaluated after 7 and 14 days of drug administration. Tissue samples for gene expression analyses in the inferior colliculus were obtained at the final time point.

The series of experiments were performed at the same time and by the same experimenters. The experimental design is shown in Figure 1. The GASH/Sal animals were divided into four groups (six animals per group): (1) GASH/Sal animals that received the vehicle (Cremophor-based) treatment (the so-called sham group); (2) GASH/Sal animals that received VPA treatment at 300 mg/kg; (3) GASH/Sal animals that received CBD treatment at 100 mg/kg; and (4) GASH/Sal animals with concomitant treatment of VPA (300 mg/kg) and CBD (100 mg/kg). Prior to the initiation of corresponding vehicle or drug treatments, all animals were subjected to the different tests (open-field and blood biochemical analyses) to measure the baseline pre-treatment parameters, and they further underwent the seizure stimulation protocol to confirm that all of them developed a complete AGS (Figure 1). The drugs used in the experiments were stored in a freezer (at ~-20°C), protected from light and freshly prepared immediately prior to injection. The VPA (Depakine®, Sanofi Aventis) has been successfully used in a previous study showing anticonvulsant effects in GASH/Sal animals at its peak plasma concentrations, which was 30 to 45 min after the intraperitoneal injections (Barrera-Bailón et al., 2013). According to this study, VPA had 75% protective effect against seizures at doses of 300 mg/kg; therefore, we precisely selected this dosage to determine possible synergistic (additive or antagonistic) effects when administered in combination with CBD. CBD, extracted from cannabis plants, was subsequently purified and provided by RiverForce Partners Inc. (Boston, MA, USA). Purity for the phytocannabinoid was >99% (according to the supplier's information certificate). The CBD powder was suspended in Cremophor® RH 40/ethanol/saline in a ratio of 1:2:17 and mixed by Vortex shaker followed by sonication. The CBD dose of 100 mg/kg was selected based on the results of a separate experiment performed in 14 GASH/Sal animals, in which we determined the timing of the maximal levels of CBD by measuring the serum concentration of the drug after a single intraperitoneal administration. The results of this pharmacokinetic evaluation are shown in Figure 3. All vehicle and drug treatments were injected intraperitoneally. To determine the effects of the corresponding acute drug administrations, all groups of animals were examined 45 min after the first intraperitoneal injection for AGS severity (Figure 1). To determine the effects of chronic drug administrations, the animals with VPA treatment received repeated intraperitoneal injections of VPA at a dosage of 300 mg/kg every 24 h for 2 weeks, the animals with CBD treatment received repeated intraperitoneal injections of CBD at a dosage of 100 mg/kg every 12 h for 2 weeks, and the animals with the combined CBD and VPA treatment received repeated intraperitoneal injections of VPA and CBD at the corresponding dosage used in the single drug treatments (Figure 1). In all animal groups at 7 days, effects of each drug treatment were evaluated for open-field test and seizure severity. Furthermore, the effects of chronic drug administrations were also evaluated in all animal groups at 14 days (the final time point) for open-field test, blood biochemical analyses, and AGS severity. All these tests for evaluating the effects of chronic drug administration were carried out 8 h after the first intraperitoneal administration of the day (at 7:30 a.m.) and in an orderly manner leaving 24 h in between. Tissue samples for gene expression analyses in the inferior colliculus were obtained at the final time point (Figure 1).

### Open-Field Test

The open-field test was used for assessment of exploratory behavior and general locomotor activity in the GASH/Sal before and after the treatments with vehicle, VPA, CBD, or both CBD and VPA (CBD+VPA) at the protocol-defined time points, detailed in Figure 1. The protocols of the open-field test were applied the same way for all treatment groups, and the experiments were conducted between 3:30 and 5:30 p.m. at the same light intensity and temperature conditions, in which the animals were housed. The handling of the animals was carried out by the same investigator, and the field was carefully cleaned with a disinfectant alcohol-based solution and thoroughly dried after each animal. The test was performed prior to the loud sound stimulation, and every GASH/Sal animal underwent video recording during every open-field assessment. The open-field box was made of white acrylic plastic with a circular arena of 80 cm in diameter and rounded by a 30 cm-high wall. The arena was subdivided into three zones: central, intermediate, and external, each occupying one third of the total surface area. At the time of testing, individual animals were removed from its home cage and immediately placed into the center of the field. The performance and paths of the animals were recorded for 10 min using a digital video camera set at 1.20 m above the ground. The ANY-maze software (Stoelting Co., v. 6.16) was used to automatically analyze the animal's locomotor and exploratory activity (time spent in activity, distance traveled, and number of rearings) and emotional performance (time spent in central zone and number of groomings).

### Blood Extraction and Sample Preparation

Sample collection and preparation were carried out following a similar procedure previously described by Barrera-Bailón et al. (2013) at protocol-defined time points, detailed in Figure 1. The blood was extracted from the cranial vena cava under inhalation anesthesia (induction: 4% isoflurane and 1 L/min O_2_; maintenance: 3% isoflurane and 0.4 L/min O_2_), immediately stored in plastic tubes, and right after obtaining the sample, equal volume of blood was replaced by receiving subcutaneous hydration of 0.9% saline. At each time point, a specific amount of blood was prepared according to the type and nature of the analysis to be carried out. The 200 μL samples of blood were drawn for measuring the CBD serum levels and kept at room temperature for 1 h and subsequently on ice during 30 min until a clot was formed. Then, these blood samples were centrifuged for 20 min at 4,000 rpm at 4°C. The supernatant (serum) was collected in aliquots of 100 μL and frozen until the pharmacokinetic analysis of CBD concentrations. For the hematological assessment, 500 μL of blood was collected in K3-EDTA–coated sample bottles and immediately analyzed after drawing each sample to prevent errors in cell counting. For the analysis of blood biochemistry, the collected volume was 400 μL that was kept in the plastic tube, without anticoagulant substances, at room temperature for 30 min until a clot was formed. Subsequently, these blood samples were centrifuged at 4°C for 10 min at 10,000 rpm, and then the serum was stored at −20°C until the moment of the biochemical analysis.

### Serum Concentrations of CBD

A separate set of experiments was initially carried out to determine the serum concentrations levels of CBD after intraperitoneal administration of 100 mg/kg. For this purpose, 14 GASH/Sal animals followed the blood extraction and preparation protocol described above at various times after administration of the CBD: 15, 30, 45, 60, 120, 240, 480, 720, and 1,440 min. The blood samples were analyzed with the high-performance liquid chromatography (HPLC)/mass spectrometry method at the Mass Spectrometry Service of the USAL. The equipment used consisted of a Waters Acquity UPLC HT® system (Waters Corporation, Milford, MA, USA) equipped with a binary pump, an online degasser, and a thermostatic column compartment for the HPLC assay and a tandem quadrupole Waters Xevo TQS micro system (Waters Corporation) for the mass spectrophotometry assay. The mobile phase consisted of A (water containing 0.1% formic acid) and B (acetonitrile) and was delivered at a flow rate of 0.7 mL/min to an Acquity UPLC BEH C-18 column (particle size: 1.7 μm, diameter: 2.1 mm, length: 50 mm) (Waters Corporation, Milford, MA, USA). The method of elution for the CBD was carried out using the following gradient program: 95% A and 60% B at 0.00 min, 90% A and 10% B at 0.35 min, 75% A and 25% B at 0.99 min, 0% A and 100% B at 1.00–1.90 min, and 95% A and 5% B at 1.91 min. The pharmacokinetic profile after CBD intraperitoneal administration is shown in Figure 3.

### Body Weight and Hematological and Biochemical Analysis

The body weight of the animals belonging to each experimental group was measured three times a week to determine the possible side effects of each treatment on weight gain, as well as on food and water intake. Hematological analysis of the blood samples was performed at the end of the chronic treatments by using an automated hematology analyzer (ADVIA® 120 Hematology System, Bayer, Germany). The hematological parameters that were evaluated included hemoglobin concentration, hematocrit, and the number of red blood cells, white blood cells, and platelets per microliter. To assess the biochemical markers of liver function, the following biochemical assays were performed in the serum samples: hepatic enzymes such as aspartate aminotransferase and alanine aminotransferase, as well as bilirubin, serum albumin, and total protein. The concentrations of those biochemical parameters were estimated using standard laboratory kits (Spotchem II Liver-1 kit, #33925, Menarini Diagnostic PAIS), as per manufacturer's instructions, and the spectrophotometric reading was made in a continuous flow system by using the automated Spotchem EZ analyzer (SP-4430). All these procedures were performed in the Experimental Animal Facility of the University of Salamanca.

### Stimulation for Sound-Induced Seizures

The AGSs were induced by intense acoustic stimulation 45 min after the acute drug administration and 8 h after the last drug administration for analyzing the chronic drug effects (Figure 1). The sound stimulation protocol for triggering AGS in the GASH/Sal animals followed the same procedure previously used by Barrera-Bailón et al. (2013, 2017). The animals were placed in a cylindrical acrylic arena (height: 50 cm, diameter: 37 cm) and allowed to acclimate for 1 min. The animals were then exposed to a continuous white noise of 0 to 18 kHz and an intensity of 115 to 120 dB, to induce AGSs. Exposure continued until the initiation of wild-running phase or until 20 s had elapsed, whichever came first. The recorded sound was created using a high-pass filter (>500 Hz, Bruel & Kjaer #4134 microphone and preamplifier #2619), digitized at 44.1 kHz, and played by a computer-coupled amplifier (Fonestar MA-25T, Revilla de Camargo, Spain) and speaker (Beyma T2010, Valencia, Spain) located above the arena.

### Video Recordings, Seizure Severity Index, and Neuroethological Analysis

**Figure 2:**
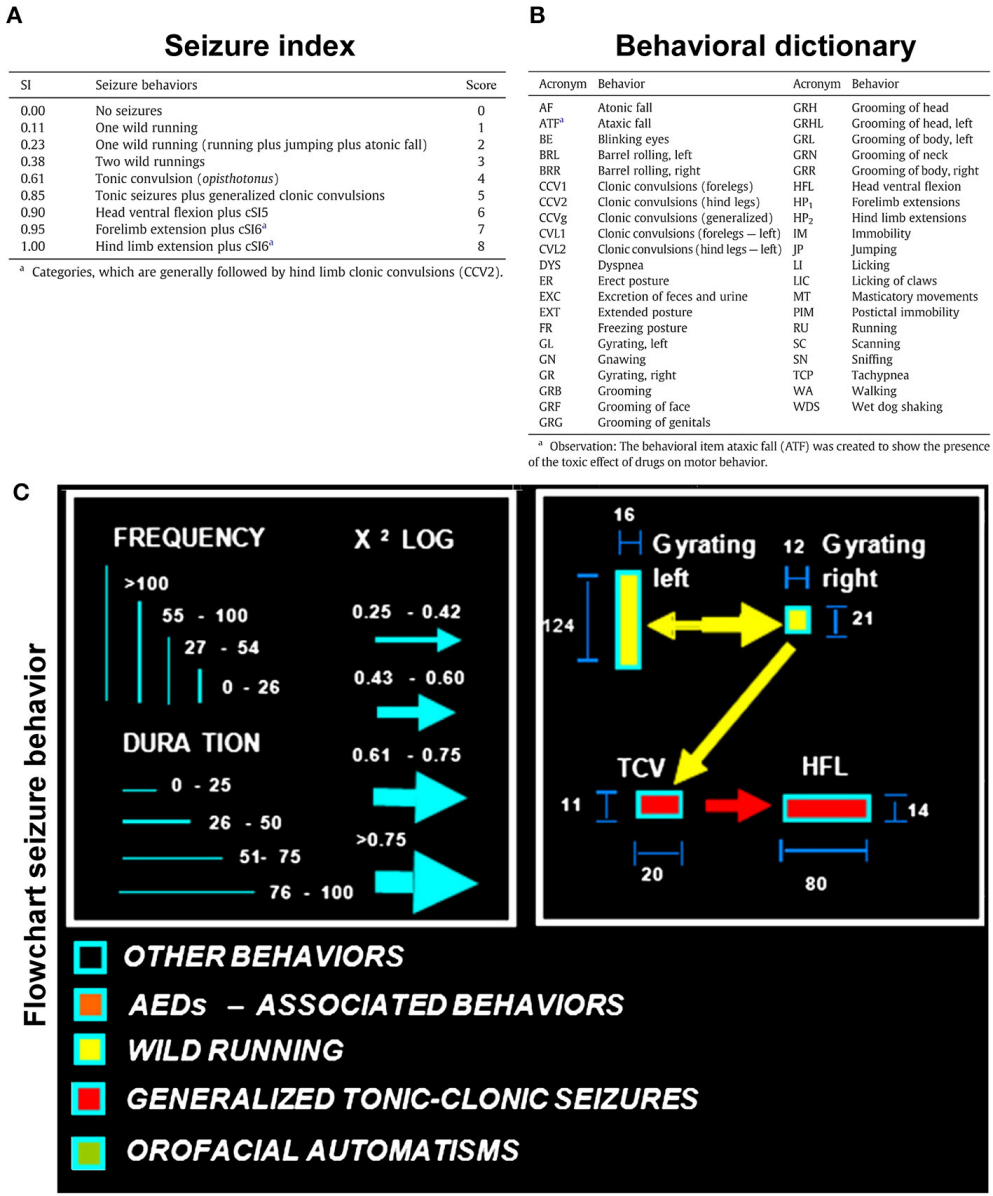
Audiogenic seizure analysis in the GASH/Sal. **(A)** Seizure index (SI), according to Garcia-Cairasco et al. (1996) using the behavioral descriptions and categorized severity scores transformed into discreet variables for statistical purposes. **(B)** Dictionary of the behavioral variables analyzed and displayed in the flowcharts of the seizure behaviors. **(C)** Flowcharts illustrating the graphical and statistical aspects of the seizure behaviors. The frequency and time spent performing each behavior are proportional to the height and width of the rectangle, respectively. The arrow width and direction indicate the statistical intensity and preference association between two behavioral items. The behaviors associated with wild-running phase (including wild running, jump, and falls) are depicted in yellow, and the behaviors associated with the convulsion phase (including convulsions and extentension of extremities) are depicted in red (figure modified from Barrera-Bailón et al., 2013).

Video recordings of seizure activity in the GASH/Sal animals began 1 min prior to the sound exposure and continued until the animal recovered from the stupor. To assess the intensity and severity of the AGS in the GASH/Sal animals, we followed the same tools and behavioral scoring previously described by Barrera-Bailón et al. (2013). Briefly, the analysis of each recording session was based on the behavioral repertoire of the animals during the seizure at three time windows: 1 min before the starting of the acoustic stimulation (presound), the first 30 s during the acoustic stimulation (sound), and 1 min after the acoustic stimulation (postsound). The severity index used to assess the overall intensity of the seizures in the GASH/Sal has been used previously (Garcia-Cairasco et al., 2004; Rossetti et al., 2006; Barrera-Bailón et al., 2013) and is presented in Figure 2A. After each animal was assessed for seizure severity, we divided the animals into two groups for further analysis. Those with a seizure severity scores ≥2 were considered as animals that maintained seizures, and those with a seizure severity scores lower than 2 were considered as animals with no seizures or very mild symptoms. The seizure latency was assessed in all animal groups and was defined as the period of time between the stimulus onset and the first behavioral manifestation of the wild-running phase. The behavioral sequences observed during the seizure phases were assessed using a neuroethological method (quantitative behavioral analysis) that provides integrative and reliable information about seizure generation, neuronal structures, and the sequential behavioral expression (Garcia-Cairasco et al., 1996, 2004). Every behavior presented in a given time window was recorded, second by second, according to a dictionary of behavioral items (Figure 2B) described by Garcia-Cairasco et al. (1996). Once the data were obtained and after reviewing the video several times, the ETHOMATIC program calculates and displays the mean frequency and mean duration of each behavioral item in the given observation window (presound, during sound, and postsound stimulation). The program also performs statistical analysis, verifying significant associations between pairs of behavioral items and calculating χ^2^ values. Flowcharts representing all of the statistically significant data were constructed using Microsoft PowerPoint 2011 (Figure 2C).

### Gene Expression Analysis (Quantitative Real-Time Polymerase Chain Reaction)

The inferior colliculi of the GASH/Sal animals were collected at the final time point of the experiments to study the differential expression of the following genes: *Trpv1, 5-Htr1a, Sigmar1, Adora1, Slc29a1*, and *Cnr1*. Those genes were selected as they encode neuronal receptors related to regulation of neuronal excitability and neuroprotection that might be presumably affected by the pharmacological activity of the CBD. The inferior colliculi of each animal were obtained following euthanasia by deep anesthetization and rapid decapitation. Each sample tissue was frozen immediately in liquid nitrogen. The reverse transcription–quantitative polymerase chain reaction (RT-qPCR) experiments were carried out following the protocol routinely used in our laboratory (López-López et al., 2017; Damasceno et al., 2020; Díaz-Rodríguez et al., 2020; Sánchez-Benito et al., 2020), which is in accordance with published guidelines (Nolan et al., 2006). Briefly, total RNA (2 μg) was mixed with oligo-dT and random hexamer primers for reverse transcription into cDNA using the First Strand cDNA Synthesis Kit (K1621, Promega Corporation, Madison, WI, USA). In all cases, a reverse transcriptase negative control was used to test genomic DNA contamination. Subsequently, qPCR was performed using the SYBR Green method with a 2× Master Mix (#4367659, Applied Biosystems). Each reaction contained 10 μL of Master Mix, 0.4 μL of each pair of primers, 3 μL of each cDNA sample in a different serial cDNA quantity for each gene, and MilliQ water (RNA-free) up to 20 μL. The amplification reaction was performed in the QuantStudio 7 Flex Real-Time PCR System (Applied Biosystems) under the following conditions: 10 min at 95°C followed by 40 cycles of 15 s at 95°C and 30 s at 60°C depending on each pair of primers. RT-qPCR experiments were performed in a set of four to six biological replicates (sample cases) for each experimental group (treatment condition) and conducted in three technical replicates from each sample and gene product examined. The list of primers used in the RT-qPCR experiments is provided in Table 1.

**Table 1:** Oligonucleotide primers used for RT-qPCR, indicating the location of each primer in the corresponding Ensembl sequences of the Syrian hamster.

**Gen target**	**ID transcript Ensembl *Mesocricetus auratus***	**Primer forward**	**Primer reverse**	**Size of products**	***E^a^***
*Trpv1*	*ENSMAUG00000008181*	GACGAGGGTGAACTGGACTACC	CTTGACACCCTCACAGTTGC	60	2.16
*5-Htr1a*	*ENSMAUG00000007970*	CACCATCAGCAAGGATCACG	AGAGCAGCAGCGGGATATAGA	85	2.03
*Sigmar1*	*ENSMAUG00000011503*	AGAGAGGGCACCACGAAAAG	AAGGAGCGGAGGGTATAGAAGA	88	1.97
*Adora1*	*ENSMAUG00000018351*	CATCGTATCCCTGGCGGTAG	ACGCAGGTGTGGAAGTAGGTCT	75	2.01
*Slc29a1*	*ENSMAUG00000006341*	CATCAGGAGGTGTGTGGGTTT	TCATGGTTCCAGGGTTCTCG	162	2.07
*Cnr1*	*ENSMAUG00000014040*	TGTTGACTTCCATGTGTTCCA	GGTCTGGTGACGATCCTCTT	126	1.98
*Actb*	*ENSMAUG00000008763*	AGCCATGTACGTAGCCATCC	ACCCTCATAGATGGGCACAG	105	2.03

a *qPCR primer efficiency (E) was calculated according to the following equation: E = 10 ^(−1/slope)^*.

A standard curve was made to verify the efficiency of the primers of the target and reference genes, and it was constructed by serial dilutions of cDNA isolated: 60, 30, 15, 7.5, 3.25, and 1.65 ng/μL. Data showed that all genes used in this work were expressed at a high level, and investigated transcripts showed high linearity (*R*^2^ > 0.95). Real-time PCR efficiencies (*E*) of one cycle in the exponential phase were calculated according to the equation *E* = 10^[−1/slope]^. High PCR efficiency rates were shown to occur in the investigated range of nanogram cDNA input, and all genes produced approximately identical slopes. To determine which was the most stable reference gene for RT-qPCR data normalization, two candidates [β-actin (*Actb*) and glyceraldehyde 3-phosphate dehydrogenase (*Gapdh*)] were selected, and their expression was measured by NormFinder software (Andersen et al., 2004) that calculate intragroup and intergroup variations in gene expression. Thus, the mean threshold cycle (Ct) value and primer efficiency value of *Actb* were used for data normalization. The comparative Ct method was used for quantitative data analysis (Schmittgen and Livak, 2008). After removing outliers (Burns et al., 2005), the relative gene expression value of each transcript was calculated according to the formula 2^−(Δ*Ct“*condition 1^^”^^−Δ*Ct“*condition 2^^”)^, where “condition 1” corresponds to the experimental sample, “condition 2” to the sample from the control animal, and the ΔCt of each “condition” is Ct_“experimentalgene”_ – Ct_“endogenousgene”_ (Schmittgen and Livak, 2008).

### Statistical Analysis

Statistical analyses including the scoring of seizures and relative gene expression values were performed using the SPSS-IBM software, version 25.0 (SPSS Inc., Chicago, IL, USA). All quantitative data were expressed as mean value ± standard error of the mean (SEM). Statistical analyses corresponding to the seizure behaviors, body weight, and hematological and biochemical parameters were performed using two-way mixed analysis of variance (ANOVA) with Bonferroni *post hoc* test. Statistical analyses for the open-field behavior and the mRNA expression levels were conducted using one-way ANOVA followed by Bonferroni multiple-comparisons test. The differences between experimental groups were considered statistically significant with *p* < 0.05 (^*^), *p* < 0.01 (^**^), and *p* < 0.001 (^***^). All the data were plotted using GraphPad Prism (version 6.05).

## Results

### Pharmacokinetic Profile of CBD in Serum

**Figure 3:**
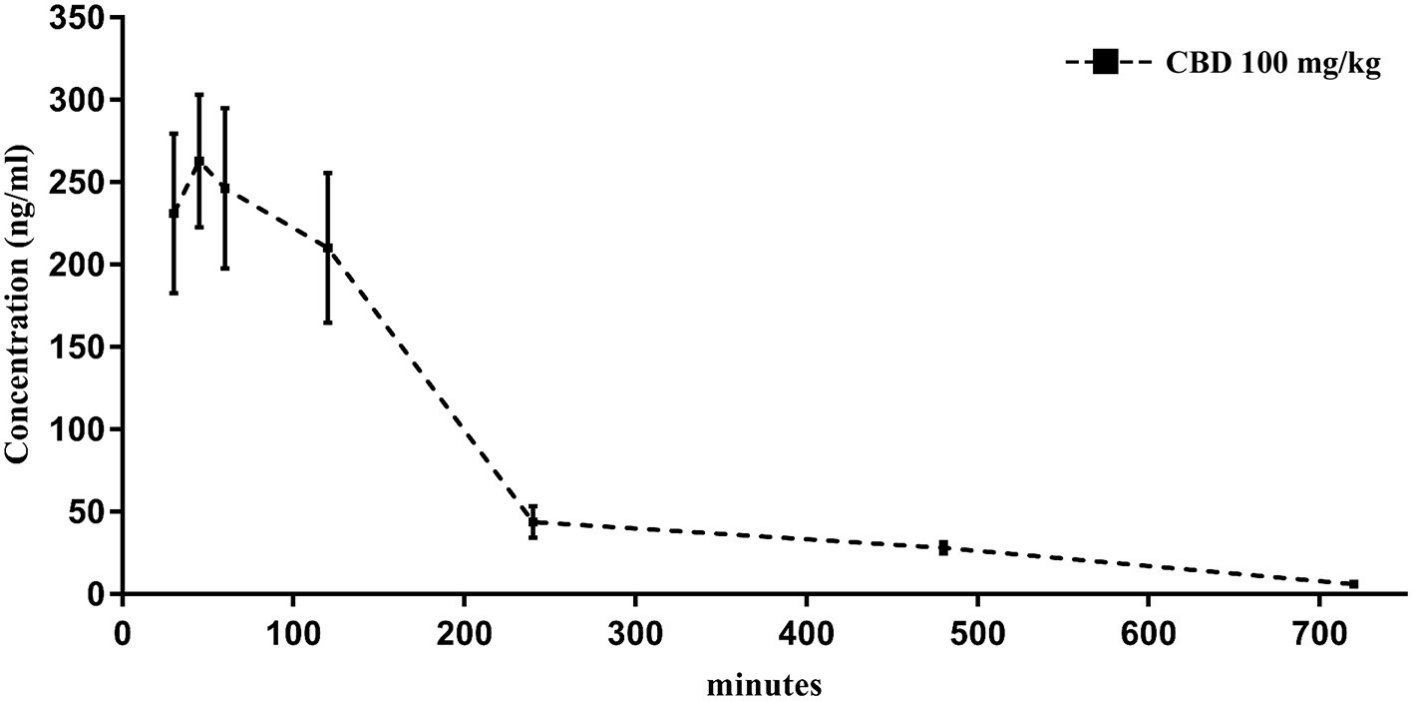
Time course of CBD levels in serum from GASH/Sal animals treated with CBD (100 mg/kg, intraperitoneal). Notice that levels of CBD peak within 45 min after the intraperitoneal injection. Results are expressed as means ± SEM (*n* = 14).

The pharmacokinetics of CBD, when given as an intraperitoneal single 100 mg/kg dose, were determined in serum of GASH/Sal animals. The mean concentration–time profile of serum for the 14 animals is illustrated in Figure 3. The highest mean serum CBD concentrations were shown at 263.3 ± 40 ng/mL (45 min after administration), which dropped to 210.2 ± 45 ng/mL at 2 h. By 4 h after dose administration, the CBD concentrations in serum remained very low 43.9 ± 9 ng/mL until hour 12. As shown in the pharmacokinetic profile, elimination of CBD in sera was measured at 24 h after the drug administration (Figure 3). The pharmacokinetic profile after CBD intraperitoneal administration indicated that 45 min postinjection was the best time point to measure the possible anticonvulsant effects of CBD.

### Effects of Drug Treatments on the Behavior and Intensity of Audiogenic Seizures

**Figure 4:**
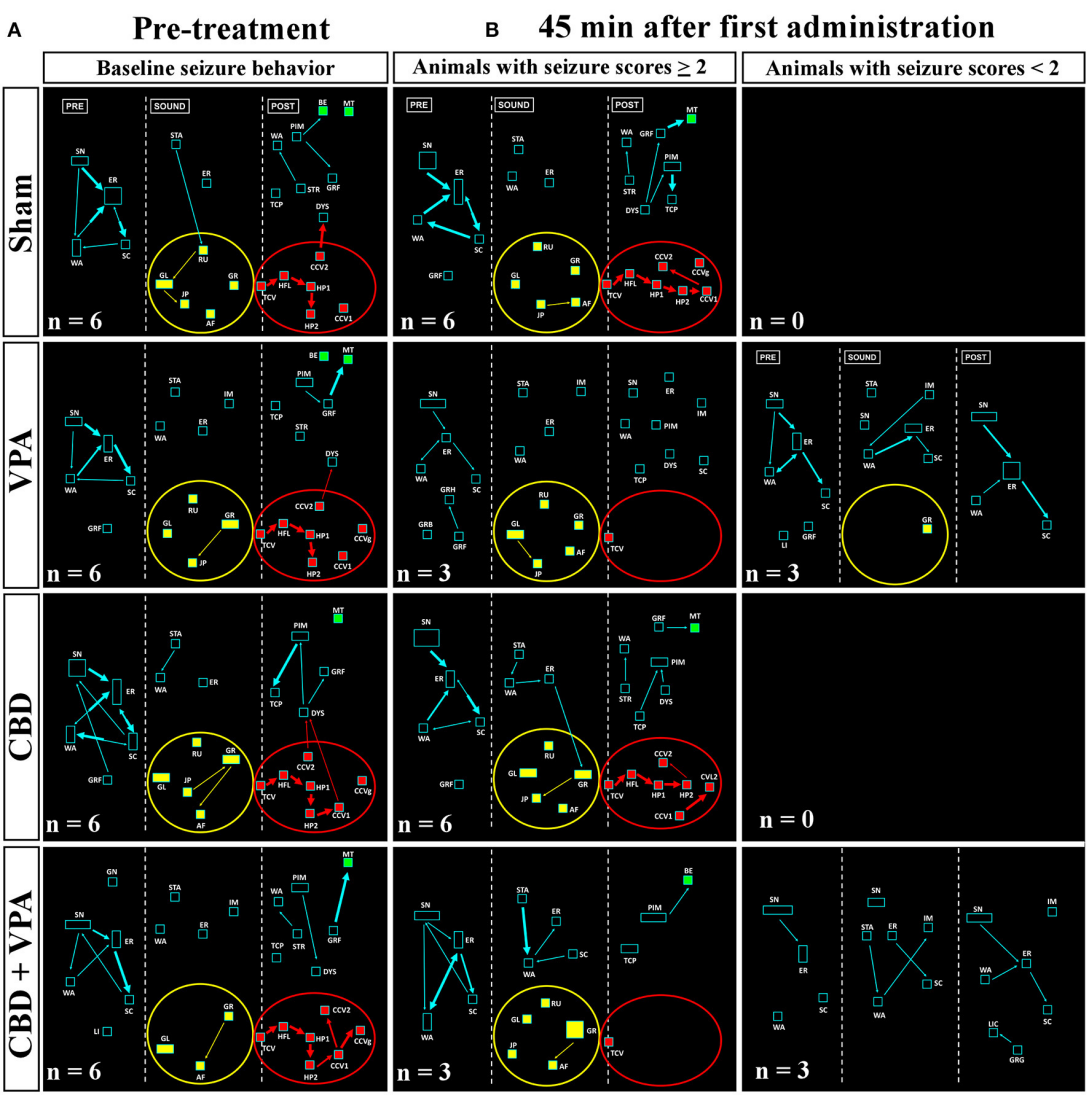
Treatment effects of VPA and CBD on the audiogenic seizure behaviors of the GASH/Sal within 45 min after the first intraperitoneal administration. **(A)** Flowcharts show the sequences of behaviors in GASH/Sal animals in baseline conditions before starting the treatment. Notice that all GASH/Sal animals exhibited a complete audiogenic seizure, including the behavioral manifestations of the wild-running phase with turns to the left (GL) and right (GR), running (RU), jumps (JP), and atonic falls (AF) (depicted together with the yellow circle), as well as the behaviors associated with tonic–clonic convulsions (TCV, CCV1, CCV2, and CCVg), followed by a behavioral cluster of generalized clonic seizures with hyperextensions (HP1 and HP2) (depicted together with the red circle) and ended with postictal immobility (PIM) and breathing difficulties [dyspnea (DYS) and tachypnea (TCP)]. **(B)** Flowchart shows the sequences of behaviors in GASH/Sal animals treated with vehicle (sham), VPA, and CBD and with a combination of CBD and VPA, 45 min after the first intraperitoneal administration. Notice that behaviors associated with tonic–clonic convulsions (red circles) were blocked with VPA—or the combined CBD+VPA treatments, whereas animals treated with vehicle or single CBD administration remained similar to baseline conditions, although the elimination of the generalized clonic convulsions (CCVg) in the single CBD administration was noticeable. Furthermore, half of the GASH/Sal animals that followed VPA treatment or the combined CBD+VPA treatments showed seizures scores <2 with almost total absence of seizure behaviors or complete blockage of seizure activity. Each flowchart showed the compendium of behaviors in all the animals belonging to each experimental group. The seizure behaviors were analyzed in three time windows (presound, during sound, and postsound). See Figure 2 for detailed interpretation of the flowchart.

**Figure 5:**
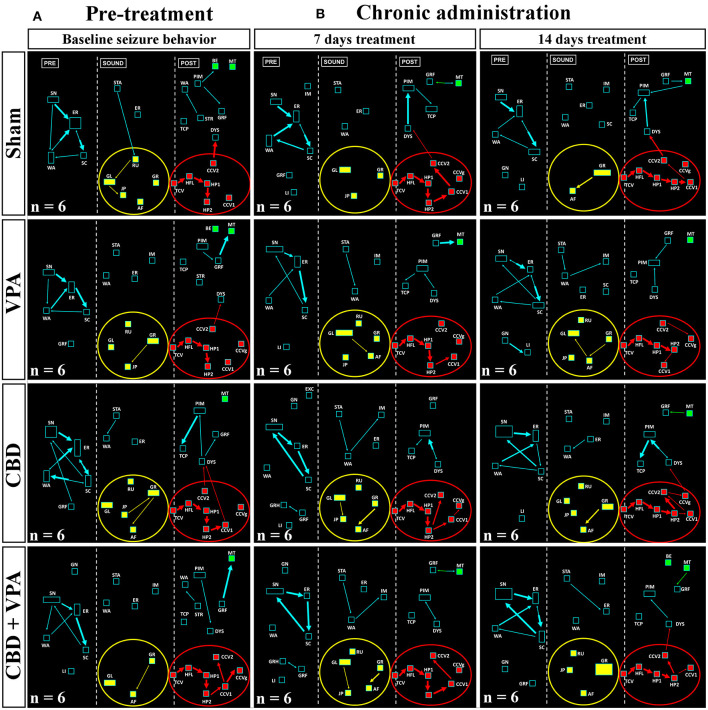
Treatment effects of VPA and CBD on the audiogenic seizure behaviors of the GASH/Sal within 7 and 14 days after chronic administration. **(A)** Flowcharts show the sequences of behaviors in GASH/Sal animals in baseline conditions before starting the treatment. Notice that all GASH/Sal animals exhibited a complete audiogenic seizure, including the behavioral manifestations of the wild-running phase with turns to the left (GL) and right (GR), running (RU), jumps (JP), and atonic falls (AF) (depicted together with the yellow circle), as well as the behaviors associated with tonic–clonic convulsions (TCV, CCV1, CCV2, and CCVg), followed by a behavioral cluster of generalized clonic seizures with hyperextensions (HP1 and HP2) (depicted together with the red circle) and ended with postictal immobility (PIM) and breathing difficulties [dyspnea (DYS) and tachypnea (TCP)]. **(B)** Flowchart shows the sequences of behaviors in GASH/Sal animals treated with vehicle (sham), VPA, CBD, and with a combination of CBD and VPA after 7 and 14 days of chronic drug administration. Notice that seizure behaviors in general remained similar to baseline conditions for all experimental groups, showing seizures scores ≥2. Notice slight modifications in the behavioral items associated with the wild-running phase, which can be seen in all experimental groups. Each flowchart showed the compendium of behaviors in all the animals belonging to each experimental group. The seizure behaviors were analyzed in three time windows (presound, during sound, and postsound). See Figure 2 for detailed interpretation of the flowchart.

**Figure 6:**
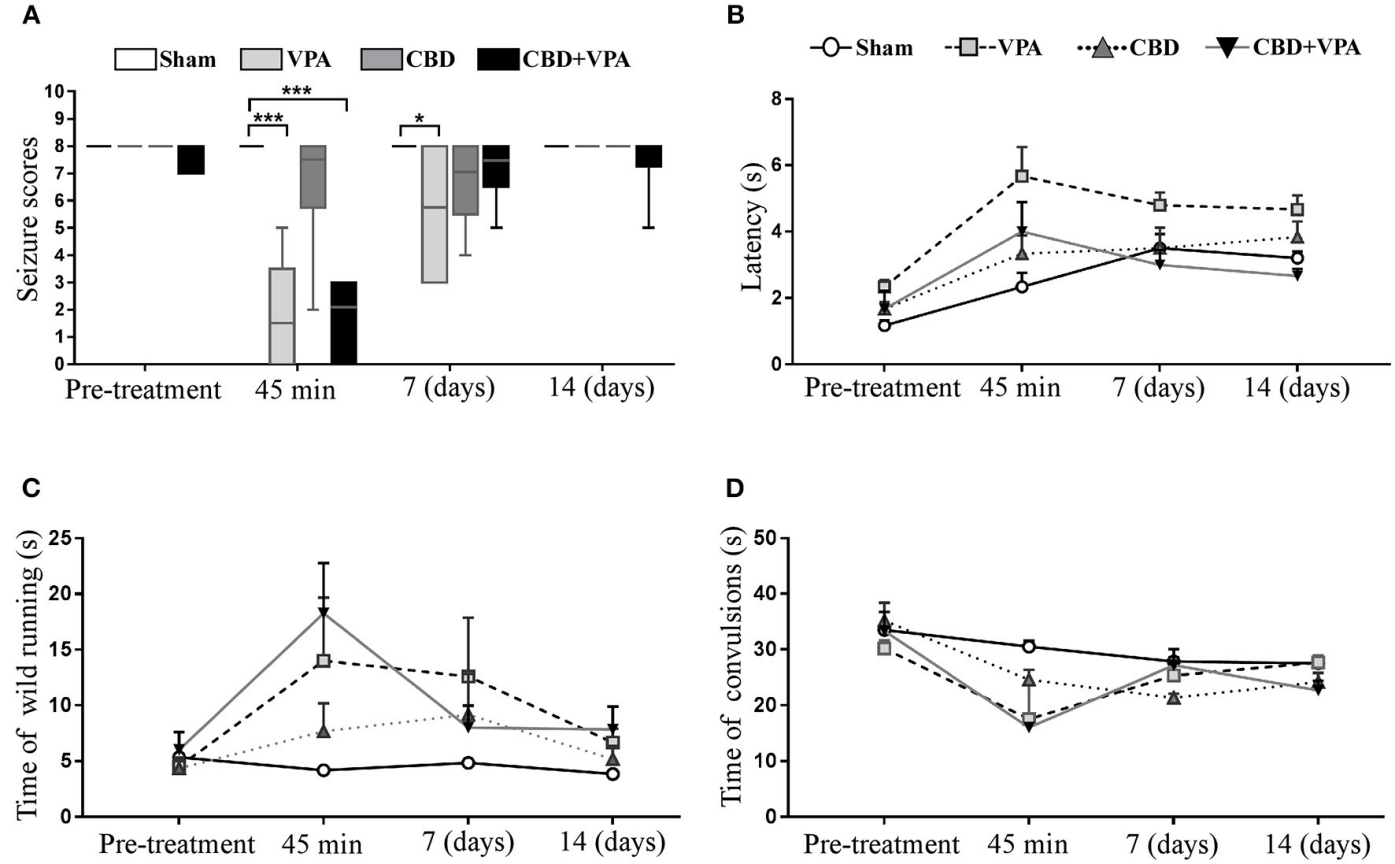
Effects of VPA and CBD on the seizure intensity, latency, and duration of wild-running and convulsion phases. **(A)** Box plot summarizing the categorized seizure-index score in each experimental group on the baseline condition (pre-treatment), after acute treatment (45 min), as well as 7 and 14 days after chronic administrations. In each box plot, the horizontal line crossing the box is the median. Asterisks indicate significant differences between experimental groups (*p < 0.05, ***p < 0.001). **(B–D)** Graphs showing the duration (in seconds) for the seizure latency **(B)**, the wild-running phase **(C)**, and the convulsion phase **(D)** in each experimental group on pre-treatment condition and 45 min, 7 days, and 14 days post-treatment. GASH/Sal animals with complete elimination of audiogenic seizures (seizure scores = 0) were not graphed in **(B–D)** as the variable time was unable to be assessed.

During loud acoustic stimulation and prior to any of the drug treatments, all GASH/Sal animals displayed a complete AGS as described by Muñoz et al. (2017). Beginning between 1 and 3 s after the stimulus onset, the GASH/Sal animals showed five differenced and consecutive phases that included (1) a behavioral arrest; (2) a wild-running period of nearly 5 s; (3) tonic–clonic convulsions for approximately 33 s; (4) head ventral flexion, forelimb, and hindlimb extensions; and (5) postictal immobility (stupor). Finally, the animals recovered walking and exploring behavior with similar features to those shown in the basal prestimulus condition. A video recording of a representative seizure in a non-treated GASH/Sal animal, together with the corresponding seizure behaviors analyzed in the ETHOMATIC program, can be simultaneously seen in Supplementary Material 1. The percentage of animals based on the achieved categorized seizures scores (≥2 or <2) is shown for each experimental group in Supplementary Material 2. Using a dictionary of behavioral items (Figure 2B) and throughout the neuroethological analysis of the AGS (Figure 2C), we confirmed that 100% of the GASH/Sal animals in the pre-treatment conditions consistently developed the seizure behaviors in a complete manner as expected (Figures 4A, 5A; Supplementary Material 1). During the presound window, the main activity of the non-treated hamsters was behavioral exploration, including sniffing (SN) and walking (WA). During the sound window, the behavioral manifestations of the wild-running phase were observed 7 s after loud sound stimulation, including turns to the left (GL) and right (GR), running (RU), jumps (JP), and atonic falls (AF). In the postsound window, the non-treated GASH/Sal exhibited tonic convulsions (TCVs), followed by a behavioral cluster of generalized clonic convulsions (CCV1, CCV2, and CCVg) with hyperextensions (HP1 and HP2) and ended with postictal immobility (PIM), as well as altered breathing [dyspnea (DYS) and tachypnea (TCP)] (Figures 4A, 5A and Supplementary Material 1). Accordingly, all GASH/Sal animals achieved the maximum scores of 8 in the categorized seizure index prior to administration of the drug treatments (Figure 6A and Supplementary Material 2) and exhibited values in seizure latency as well as in the time of the wild-running and convulsion phases that were consistent with the typical AGS in the GASH/Sal strain (Figures 6B–D). Following the experimental design, each animal group received intraperitoneal injections of either the vehicle, VPA, CBD, or the combination of both drugs (CBD+VPA) at the protocol-defined time points (acute and chronic drug administrations). In the sham group, 100% of the animals acutely and chronically treated with the vehicle exhibited behavioral manifestations of seizures that were very similar to those observed in the baseline pre-treatment condition (Figures 4, 5). In fact, all sham animals were scored with the maximum values in the categorized seizure index after both acute and chronic vehicle administrations (Figure 6A and Supplementary Material 2). After 45 min of the first intraperitoneal injection of the vehicle, the sham animals showed exploratory behaviors in the presound window, followed by the typical seizure behaviors associated with the wild-running and tonic–clonic convulsion phases in the sound and postsound windows, respectively (Figure 4B). After 7 and 14 days of the vehicle-treatment, the behaviors of the sham animals remained very similar to those observed in the acute administration and pre-treatment conditions (Figure 5B). In the VPA-treated group, the behavioral manifestation of the AGS were clearly affected at 45 min after the first intraperitoneal administration, showing complete abolition of the seizure or a significant attenuation in the behaviors associated with the wild-running and convulsion phases (Figure 4B). When compared with sham animals, the GASH/Sal a single dose administration of VPA showed at 45 min after the injection a statistically significant reduction of the seizure index score (*p* = 0.0001; Figure 6A). Thus, half of these animals injected with a single VPA dose presented seizures scores <2 and the other half exhibited very low seizure scores, which were between 2 and 5 (Figure 6A and Supplementary Material 2). After 7 days of daily repeated VPA injections, the GASH/Sal animals exhibited slight modifications of seizure behavior (Figure 5B) with a statistically significant decrease in the seizures scores as compared to sham animals (*p* = 0.03; Figure 6A). After 14 days of VPA treatment, there were not significant changes in the seizures scores with no apparent modification of seizure behaviors (Figures 5B, 6A). In the CBD-treated group, 45 min after the first administration and 7 days of repeated CBD administration produced slight and not significant attenuation of seizure behavior with seizures scores that varied between 2 to 8 (Figure 6A). In those animals acutely treated with CBD, it was noticeable the elimination of the generalized clonic convulsions (CCVg), whereas the convulsions in the legs were kept slightly visible (CCV1 and CCV2) (Figure 4B). On the contrary, 14 days of repeated daily administration of CBD had no significant effects on seizure behavior, showing maximum seizure scores in 100% of the animals (Figures 5B, 6A and Supplementary Material 2). Acute treatment with the combination of CBD and VPA drastically attenuated or completely eliminated the seizure behaviors (Figure 4B), showing a statistically significant reduction of the seizure index score when compared to sham animals (*p* = 0.0001; Figure 6A). Thus, animals acutely treated with the combination of both drugs displayed seizure scores as low as 0-1 for one half of the animals and 2–3 for the other half (Figure 6A and Supplementary Material 2). Following loud sound stimulation, GASH/Sal animals that were acutely treated with CBD+VPA and showed complete abolition of seizures exhibited normal acoustic startle reflex, exploratory behaviors and grooming (Figure 4B). Chronic administration of CBD and VPA (7 and 14 days post-treatment) produced very mild modifications of seizure behaviors associated with the wild-running phase as well as a subtle and not significant reduction in the seizure scores in 33 and 16% of the animals, respectively (Figure 6A).

### Effects of Drug Treatments on Seizure Latency and Duration of Seizure Phases

The seizure latency of sham animals followed a tendency to increase after acute and chronic vehicle-treatment (Figure 6B). Such upward trend in seizure latency was also observed in all animal groups over the experimental timeline, and hence the sham group was considered the reference for comparisons of seizure latency between groups. After 45 min of the single dose administration, we observed a significant increase in seizure latency in the VPA-treated group (5.6 s) as well as in the group with the combined drug treatment (4.0 s) as compared to the sham group (2.3 s) (Figure 6B). Animals with acute CBD treatment also showed an increase seizure latency (3.3 s), but without statistically significance as compared to sham animals (Figure 6B). Although none of the experimental groups showed a significant modification of the seizure latency after 7 and 14 days of treatment, we found an increase of ~1.8 s in the VPA-treated group, whereas the groups treated with either CBD or the combination of CBD and VPA showed the same latency than the sham group.

After acute drug administration, the duration of the wild-running phase was 14, 7.6, and 18.2 s in the VPA–, CBD–, and CBD+VPA-treated groups, respectively, which represented a significant increase when compared to sham animals (4.1 s) (Figure 6C). On the contrary, the duration of the convulsion phase was significantly reduced (17.5 s for VPA–, 24.6 s for CBD- and 16 s for CBD+VPA-treatment) when compared to sham animals (30.5 s) (Figure 6D). Therefore, the seizure latency and duration of the wild-running phase after any of the treatments in acute administration was significantly increased, whereas the duration of the convulsions were significantly reduced as compared to sham animals. These results were consistent with an attenuated effect of either acute treatment in the seizure severity of the GASH/Sal animals, in which seizures were not entirely blocked. Similar effects on the duration of the wild-running and convulsion phases were observed after 7 and 14 days of chronic drug administrations in all treatment groups, showing an increased duration of the wild-running phase and a decreased duration of convulsions as compared to sham animals, although these differences were not statistically significant (Figures 6C,D). It is noteworthy that the duration of the convulsion phase in GASH/Sal animals after 7 days of repeated daily CBD administration were significantly reduced when compared to the pre-treatment condition (*p* = 0.0001; Figure 6D). Furthermore, animals treated with 14 days administration of either CBD or the combination of CBD and VPA showed a significant reduction in the duration of convulsions as compared to their baseline condition (*p* = 0.0001 for CBD, and *p* = 0.0001 for CBD+VPA; Figure 6D).

### Effects of Drug Treatments on Body Weight and Hematological and Serum Biochemical Profiles

**Figure 7:**
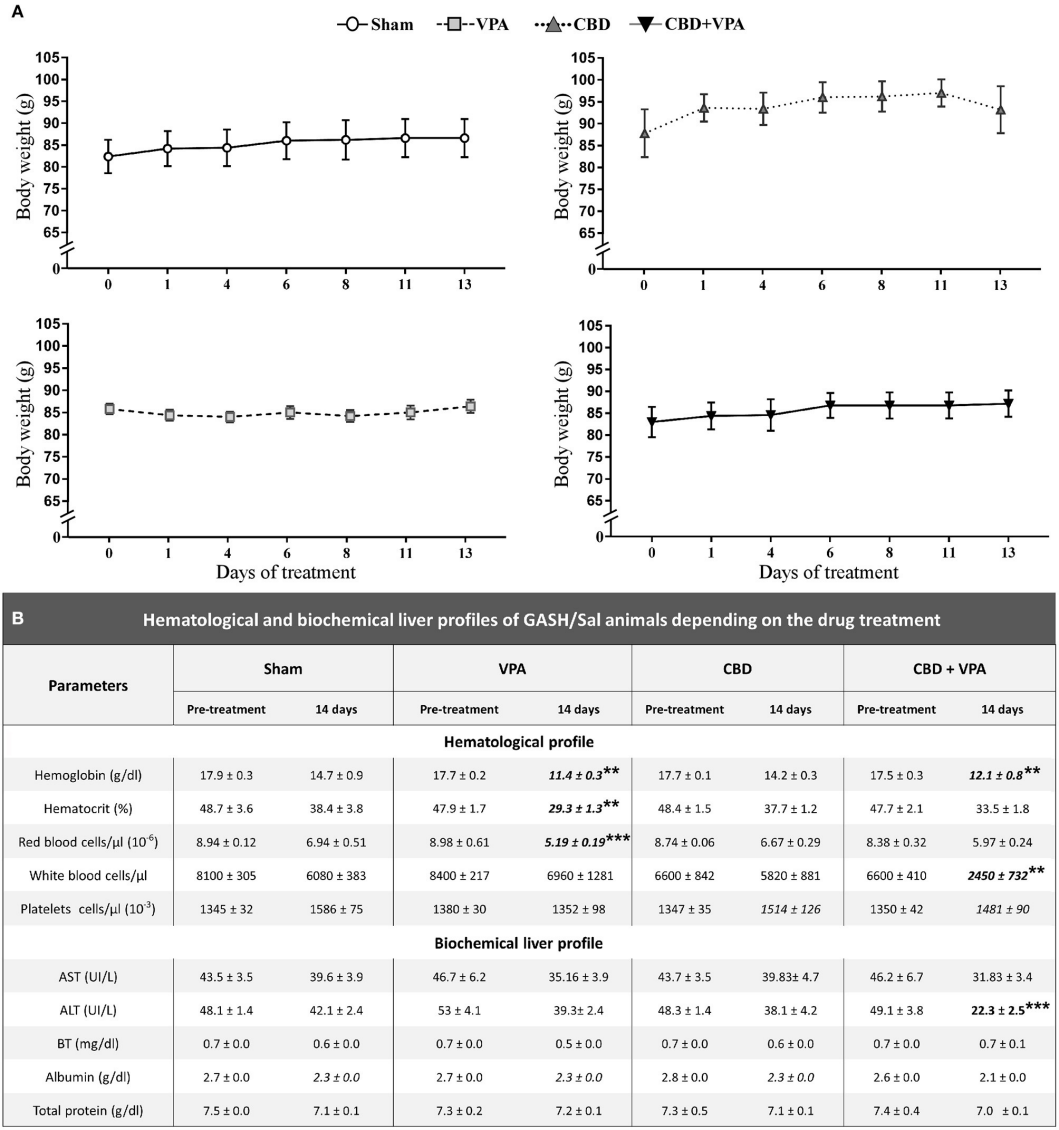
Effects on body weight and hematological and biochemical profiles of treated GASH/Sal animals. **(A)** Graphs show the time course of body weight variations monitored during the 14-day treatment in sham-, VPA–, CBD–, and CBD+VPA-treated groups. Notice that all experimental groups showed a steady and persistent body weight throughout the treatment, and no significant differences were found as compared to the pre-treatment conditions (time 0 in the horizontal axis of the graphs). Data are shown as means ± SEM. **(B)** Table shows hematological and biochemical profiles of GASH/Sal animals after 14 days of chronic drug treatments. The hematological assessment of blood included the following parameters: hemoglobin concentration, hematocrit (volume percentage of red blood cells in blood), and number of red blood cells, white blood cells, and platelets in a microliter (μL). The analysis of liver function included five biochemical parameters: aspartate aminotransferase (AST), alanine aminotransferase (ALT), bilirubin (BT), albumin, and total protein. Asterisks and bold values in the table denote statistical significance (**p < 0.01, ***p < 0.001) as compared to the pre-treatment conditions and the sham group.

We further analyzed the effects of each chronic drug administration (14 days post-treatment) on the body weight as well as on the hematological and biochemical profiles of each experimental group (Figure 7). The GASH/Sal animals showed a steady weight after chronic administration of any of the treatments and no statistically significant differences were found when compared to the baseline pre-treatment conditions. The average growth was 2.03 g/week for the Sham group, 0.30 g/week for the VPA-treated group, 3.83 g/week for the CBD-treated group and 1.96 g/week for the combined CBD+VPA treatment, showing no statistical significant differences between them and indicating normal feeding behaviors (Figure 7A). In the pre-treatment conditions, there were no significant differences between the experimental groups in any of the parameters associated with the hematological and biochemical profiles. After 14 days of chronic administration, all the hematological parameters, with the exception of the platelets, showed a statistically significant reduction in serum concentration as compared to the pre-treatment condition. Since we observed similar modifications in sham animals when compared to pre-treatment and 14 days post-treatment conditions, the sham group was considered the reference for comparisons between the drug-treated groups. Hemoglobin level decreased significantly after 14 days of chronic administration in VPA- and CBD+VPA- treated groups as compared to sham animals (*p* = 0.006; Figure 7B). In the same way, the hematocrit level was significantly lower in VPA-treated animals as compared to sham animals (*p* = 0.001; Figure 7B). Although, there were no significant differences in hematocrit level between the CBD+VPA-treated and the vehicle-treated groups, the hematocrit levels were considerable lower after co-administration of CBD and VPA, even below the reference value in the pre-treatment condition. The same effects were observed in red blood cell count, which were significantly decreased in the VPA-treated group as compared to sham animals (*p* = 0.0002; Figure 7B). Neither the CBD-treated group nor CBD+VPA-treated group showed significant differences in red blood cell count when compared to the sham group. The GASH/Sal animals treated with the combined CBD+VPA therapy showed a significant lower white blood cell count as compared to sham animals (*p* = 0.002; Figure 7B). On the contrary, neither the VPA- nor the CBD-treated groups showed significant differences in white cell counts compared to the sham group. The mean value of five biochemical parameters of liver function: aspartate aminotransferase, alanine aminotransferase, bilirubin, albumin and total protein were also assessed after 14 days of chronic administration (Figure 7B). All these serum biochemistry parameters, with the exception of the alanine aminotransferase, showed normal values very similar to those observed in the pre-treatment conditions. Thus, there were no statistically significant differences in serum levels of aspartate aminotransferase, bilirubin, albumin and total protein between all experimental groups (Figure 7B). However, the mean levels of alanine aminotransferase decreased by 55% in the CBD+VPA-treated group, showing a statistically significant lower levels as compared to the pre-treatment condition and the sham group (*p* = 0.0004; Figure 7B).

### Behavioral Characteristics According to the Responsiveness to the Drug Treatments

**Figure 8:**
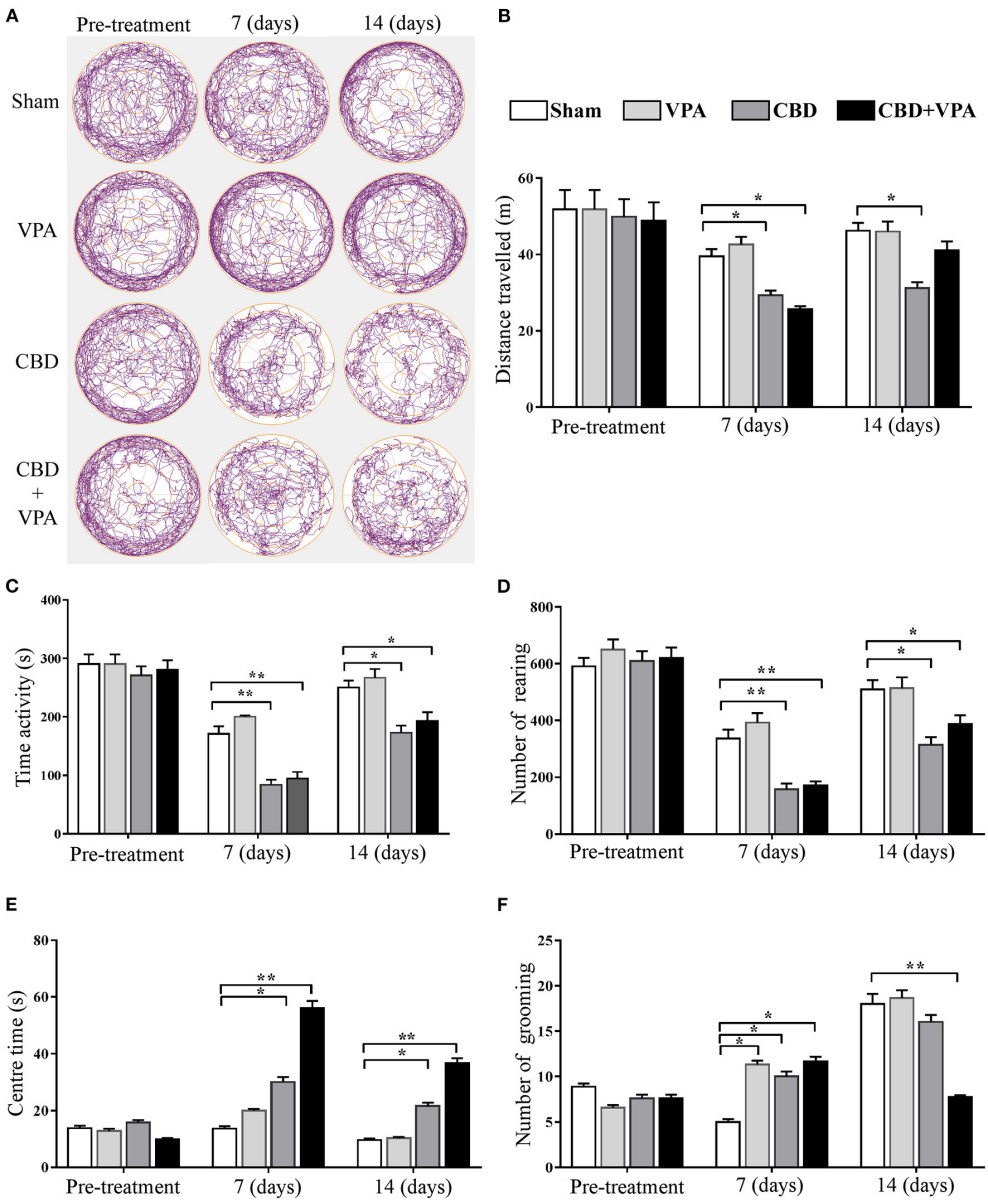
Treatment effects of VPA and CBD on the behavior of the GASH/Sal in the open-field test. **(A)** Plots show representative examples of the animal's track in each experimental group at the protocol-defined time points (pre-treatment and 7 and 14 day drug treatments). Note that levels of global locomotor activity decrease in GASH/Sal animals that received CBD alone or the combination of CBD and VPA after 7 and 14 days of treatment. Notice the trace images of GASH/Sal animals treated with CBD or CBD+VPA that spent less time moving in the periphery and longer time in the center of the arena compared to those animals treated with the vehicle or VPA alone, also compared to the pre-treatment condition. Histograms show the total distance traveled **(B)**, time of activity **(C)**, number of rearing **(D)**, time spent in the center **(E)**, and number of grooming **(F)** for all experimental groups at the protocol-defined time points. Behavior indicators for the locomotor pattern (total distance traveled, time of activity, and number of rearings) and for the emotionality (time in the central zone and number of self-grooming) revealed differential effects of CBD in both monotherapy and combined therapy with VPA when compared to baseline activity (pre-treatment), as well as to vehicle and VPA monotherapies. Data are shown as means ± SEM. Asterisks indicate significant differences between experimental groups (*p < 0.05, **p < 0.01).

We examined the VPA and CBD effects on the locomotor activity and emotionality in each experimental group by comparing automatically-scored behaviors in the open-field test after chronic drug administrations (Figure 8). The total distance traveled, time of activity, and number of rearing were used to assess general locomotor and exploratory behaviors, whereas the time spent in the center and number of grooming further provided information on emotional reactivity (i.e., fear or anxiety-like behavior). In the pre-treatment conditions, we did not observe any statistically significant differences in all GASH/Sal animals for each of the open-field measures (Figure 8). A representative example of the animal's track in each experimental group at the protocol-defined time points (pre-treatment and 7 and 14 days post-treatment) is shown in Figure 8A. Regarding the exploratory behavior and general activity, we found that the total distance traveled by the animals was significantly reduced in the GASH/Sal animals receiving either single CBD treatment or the combined CBD and VPA treatments after chronic drug administration (7 and 14 days), when compared with the sham group (Figures 8A,B). Furthermore, the administration of either single CBD treatment or combined CBD and VPA treatments drastically decreased the time of activity at 7 and 14 days post-treatment, as compared to the sham group (Figure 8C). Similarly, the total number of rearings was significantly decreased in the animal groups that received CBD or the combination of CBD and VPA after 7 and 14 days of repeated daily administration, when compared with the vehicle-treated group (Figure 8D). Such drastic reduction in general locomotor and exploratory behaviors was more evident at 7 days of repeated daily administration than that at 14 days post-treatment. At 7 and 14 days of repeated daily administration, we did not observe any statistically significant effects of single-VPA treatment on the three open-field measures related to general locomotor and exploratory behaviors (Figures 8B–D), showing similar results to those observed in the sham group. It is important to note that after 2 weeks of the combined CBD and VPA treatment the animals exhibited increased levels of locomotion when compared to GASH/Sal animal with the CBD treatment alone. We further assessed the effects of each drug treatment on emotionality by analyzing time spent in the center area and the number of groomings (Figure 8E). Compared to the sham group, both CBD alone and the combination of CBD and VPA significantly increased the time spent in the center area at 7 and 14 days of chronic administrations. Such increase in time spent in the center area was particularly noticeable in animals with the combined CBD+VPA treatments. On the other hand, there were no significant differences in the time spent in the center zone between VPA-treated and the sham groups after 7 and 4 days of chronic VPA administration (Figure 8E). After 7 days of repeated administration of the drugs, a significant increase in the number of groomings was found in the VPA–, CBD–, and CBD+VPA-treated groups as compared to sham animals. After 14 days of chronic drug administration, a drastic reduction in the number of groomings of GASH/Sal animals treated with the combined CBD+VPA treatment was noticeable, which was significantly lower as compared to sham animals. However, the animals that received the VPA or CBD treatment alone exhibited similar values in the number of grooming than those receiving the sham “vehicle” treatment (Figure 8F). The comparison between the pre-treatment and post-treatment (7 and 14 days) conditions showed a decrease in all the locomotor open-field measures, except for the time spent in the center zone that was significantly higher following the combined CBD+VPA treatment after 7 and 14 days.

### Disruption of Gene Expression in the Inferior Colliculus After Chronic Drug Treatments

**Figure 9:**
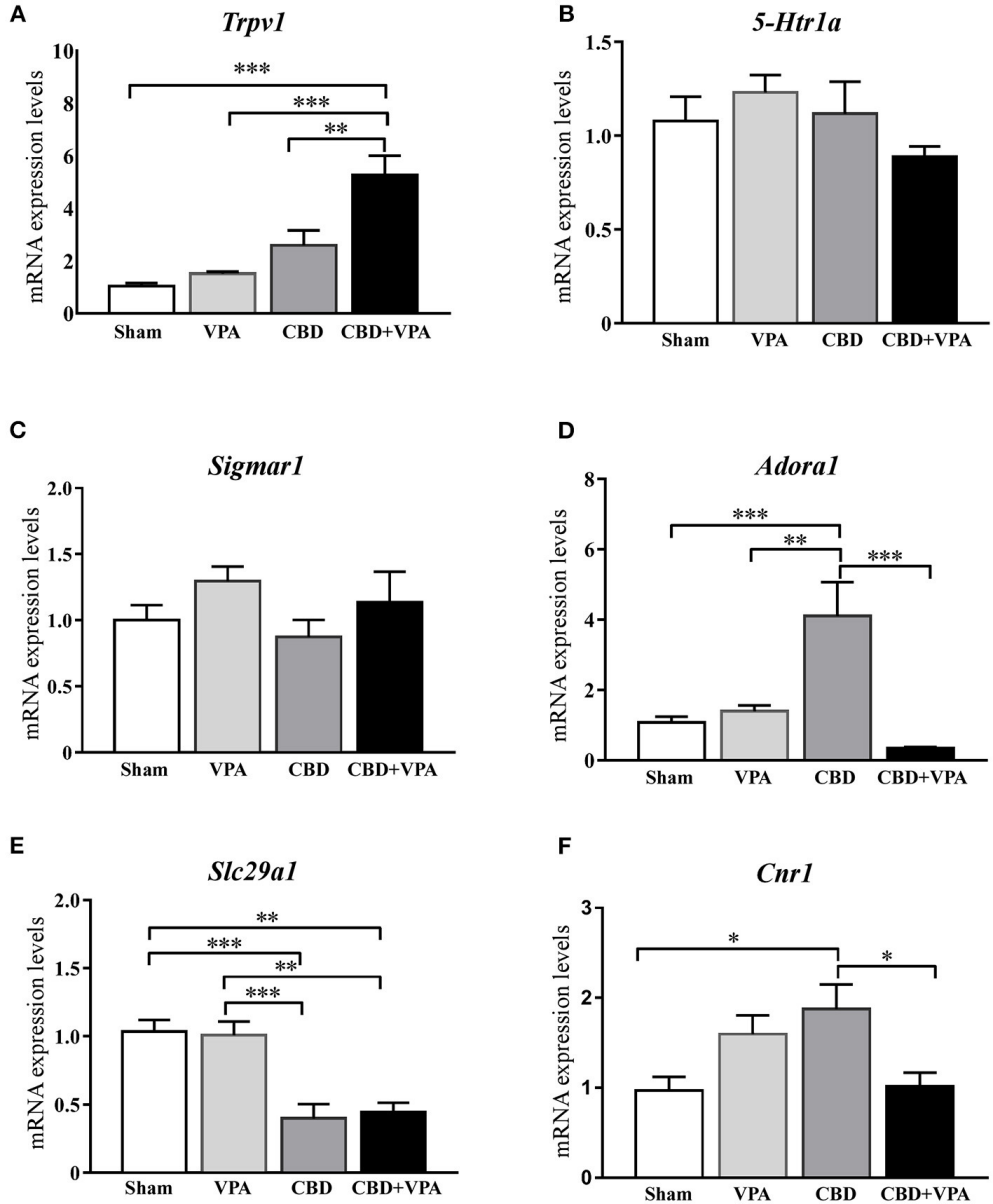
Gene expression changes in the inferior colliculus after 14-days treatment with VPA, CBD or the combination of CBD+VPA. Histogram shows relative quantities of transcripts of *Trpv1* **(A)**, *5-Htr1a* **(B)**, *Sigmar1* **(C)**, *Adora1* **(D)**, *Slc29a1* **(E)**, and *Cnr1* **(F)** genes in the inferior colliculus of GASH/Sal animals treated with vehicle (sham), VPA, CBD, and CBD+VPA. Notice that disruption of mRNA expression levels was found for the *Trpv1, Adora1, Slc29a1*, and *Cnr1* genes, whereas no differential gene expression was found for *Htr1a* and *Sigmar1*. The relative mRNA expression of each gene was normalized to β-*actin*. ΔCt values were normalized to the average ΔCt of the inferior colliculus of sham animals. Each bar in the histograms is an average ± SEM. Asterisks indicate significant differences between experimental groups (*p < 0.05, **p < 0.01, ***p < 0.001).

To determine the molecular effects of each chronic treatment on the gene expression that might underlie the modifications of seizure behaviors in the GASH/Sal, we analyzed mRNA expression levels of the following genes: *Trpv1, 5-Htr1a, Sigmar1, Adora1, Slc29a1*, and *Cnr1* (Figure 9). We selected these genes, as the proteins they encoded, are related to regulation of neuronal excitability and neuroprotection that might be presumably affected by the pharmacological activity of the treatments in the epileptogenic focus. Thus, whole tissue containing the inferior colliculi was freshly dissected after 14 days of chronic treatments from animals belonging to all experimental groups. Quantitative gene expression data were normalized using β-*actin* as internal reference gene. There were no significant differences in the number of cycles to reach the amplification threshold for *Actb* with any of the animal groups, indicating that the sample preparation was consistent. Raw gene expression data can be found in Supplementary Material 3. Comparison of gene expression/*Actb* ratios showed that expression of the *Trpv1* gene was significantly higher in the CBD+VPA-treated group as compared to the sham group (*p* = 0.0001; Figure 9A). Furthermore, the mRNA expression levels of *Trpv1* were also significantly higher in animals receiving the combination of both drugs than in those that received the CBD or the VPA treatment alone (*p* = 0.002 for CBD and *p* = 0.0001 for VPA; Figure 9A). As compared to the sham group, the gene expression of *Trpv1* was also increased with the CBD or the VPA treatment alone, although this difference was not statistically significant (Figure 9A). On the other hand, the mRNA expression levels of the *5-Htr1a* and *Sigmar1* genes were not significantly different between the four experimental groups (Figures 9B,C). CBD treatment induced modifications in the mRNA expression levels of *Adora1*, showing a significant increase as compared to the vehicle-, VPA-, and CBD+VPA-treated groups (*p* = 0.0002 for the vehicle, *p* = 0.001 for VPA, *p* = 0.0001 for CBD+VPA; Figure 9D). VPA either alone or in combination with CBD did not affect the gene expression of *Adora1* (Figure 9D). As compared to sham animals, gene expression levels of *Slc29a1* were also found significantly decreased in the animals treated with CBD alone or the combination of CBD and VPA (*p* = 0.0004 for CBD, *p* = 0.001 for CBD+VPA; Figure 9E). However, there were no significant differences in the expression levels of the *Slc29a1* gene between the VPA-treated and the sham groups (Figure 9E). Compared to sham animals, mRNA expression levels of *Cnr1* were significantly increased after the CBD treatment (*p* = 0.02; Figure 9F), whereas no significant differences were found after the VPA and VPA+CBD treatments, albeit at still high levels. Furthermore, treatment with CBD alone significantly increased the mRNA expression levels of *Cnr1* as compared to animals that received the combined CBD+VPA treatment (*p* = 0.04; Figure 9F).

## Discussion

The GASH/Sal model has been previously used to evaluate the specific efficacies of antiepileptic drugs against generalized tonic–clonic seizures. The fact that classic anticonvulsant drugs such as valproate (VPA), phenobarbital, and lamotrigine were highly effective in suppressing sound-induced seizures in the GASH/Sal indicates that this animal model can be reliably used for preclinical screening of novel anticonvulsant agents (Barrera-Bailón et al., 2013, 2017). Consistent with previous reports (Barrera-Bailón et al., 2013), we reported that acute administration of VPA (300 mg/kg) had clear anticonvulsant effects, showing that 50% of the GASH/Sal animals entirely blocked their sound-induced seizures. These results were consistent with previous studies in the GASH/Sal (Barrera-Bailón et al., 2013), in which the median effective dose of VPA for complete elimination of AGS was 300 mg/kg. We further reported that 7 and 14 days of two times intraperitoneal daily injections of VPA at doses of 300 mg/kg diminished its effectiveness in seizure attenuation when compared to an acute single-dose administration. This loss of anticonvulsant activity after chronic administration of VPA (three times intraperitoneal daily injections at 200 mg/kg for 6 weeks) was also reported in amygdala-kindled rats, showing ataxia and muscle relaxation as the most prominent adverse side effects (Hönack and Löscher, 1995; Löscher and Hönack, 1995). Although we did not find a significant impairment of locomotor exploratory behaviors after chronic VPA treatment, previous studies from Barrera-Bailón et al. (2013) also reported ataxic effects of VPA at doses of 300 mg/kg, which is in line with the reported side effects of VPA (Löscher and Hönack, 1995). Other currently available antiepileptic drugs, such as VPA, are also known to produce a variety of psychiatric, cognitive, and motor adverse effects (Zaccara et al., 2004). Therefore, therapies with similar anticonvulsant efficacy but lower toxicity or that carries the potential to reduce the adverse effects of classical antiepileptic drugs are a rapidly increasing clinical demand that has to be met. Among the novel anticonvulsant agents, there is considerable interest in CBD as a promising therapy for epilepsy, and preliminary experiments appear to demonstrate that CBD may be a useful treatment for pharmacoresistant epilepsy (reviewed in Perucca, 2017; Franco and Perucca, 2019; Lazarini-Lopes et al., 2020). Although it is also true that CBD does not exert seizure-protective activity in the rat model of pharmacoresistant epilepsy, namely, lamotrigine-resistant amygdala kindled rat (Klein et al., 2017), there is ample evidence that CBD effectively does it in a large variety of preclinical seizures models (Franco and Perucca, 2019; Patra et al., 2019; Lazarini-Lopes et al., 2020). The seizure rodent models, in which CBD was an effective and relatively potent anticonvulsant, are those induced by a great number of experimental treatments such as maximal electroshock, pentylenetetrazole, 3-mercaptopropionic acid, bicuculline, picrotoxin, cocaine, and isoniazid, as well as pilocarpine models of temporal lobe seizures among many others (Perucca, 2017; Patra et al., 2019; Lazarini-Lopes et al., 2020). The differences in the CBD effects on preventing seizures varied not only depending on the animal seizure model, but also in the dosages and route of administrations used in each study. Nevertheless, despite those differences, there is a consensus in the scientific literature that CBD given intraperitoneally produced a dose-dependent protection against seizures induced by chemical or electrical means, in acute and, more particularly, kindling rodent seizure models (Klein et al., 2017; Patra et al., 2019; Lazarini-Lopes et al., 2020). On the contrary, there are considerable fewer studies that investigated the effects of CBD on AGS models of genetic origin (reviewed in Lazarini-Lopes et al., 2020). In AGS susceptible rats, the median effective dose of CBD that reduced generalized tonic–clonic seizure was 82 mg/kg following acute intraperitoneal administration (Consroe and Wolkin, 1977). In susceptible DBA/2J mice, concomitant intraperitoneal administration of CBD (27 mg/kg) and its analog cannabidivarin (116 mg/kg) was found to attenuate wild-running, clonic, and tonic seizure, whereas isolated CBD administration was able to attenuate only the incidence of clonic seizures (Hill et al., 2013). In these few studies conducted in animal models of acute AGSs, the anticonvulsant effects of CBD were not as clear as reported in other rodent models of epilepsy. As no additional information was available from these studies about the seizure behavior analysis of the CBD effects, there is still room to investigate the potential anticonvulsant activity in genetically seizure-prone strains of rodents. Our study used neuroethological approaches in the GASH/Sal model to detail the effects of CBD on each specific seizure behavior following acute and chronic intraperitoneal administration. Our results showed that acute administration of CBD (100 mg/kg) had slight effects on seizure behaviors of GASH/Sal animals, including reduction of generalized clonic convulsions, but without significant attenuation of other behavioral items of the wild-running and convulsion phases. This behavioral CBD effects correlated to categorized index seizures scores that varied between 2 and 8, and hence none of the animals belonging to the CBD-treated group showed total elimination of seizures. Notwithstanding that, we found that acute CBD administration in the GASH/Sal significantly increased seizure latency and duration of the wild-running phase, whereas the duration of the convulsions was significantly reduced as compared to sham animals. Similar results were previously shown in other rodent models of epilepsy after CBD (100 mg/kg) intraperitoneal injections, reporting such effects on the duration of the seizure phases as a strong indication of anticonvulsant activity (Jones et al., 2012; Pelz et al., 2017). Indeed, we also observed these same effects in the GASH/Sal after acute treatment with VPA, but far more effectively and intensively than in the CBD-treated group. We further reported similar CBD effects following chronic administration (2 times intraperitoneal daily injections at 100 mg/kg) for 7 days, but in contrast, continuing this treatment over 14 days had no significant effects on seizure behavior, showing maximum seizure scores in 100% of the animals. On the basis of these results, it does not seem that CBD has potent anticonvulsant effects in the GASH/Sal model at the doses used and make us therefore discuss this matter as well as the limitations of our study. One possibility is that CBD preparation of 100 mg/kg was not therapeutically significant as it might not be the one required to reach higher CBD concentration in serum and brain. CBD levels in blood and brain may vary depending on the drug purity and concentration, solvent, and route of administration, as well as the animal species and the own characteristics of each animal model. In our experiments, we used high purity of CBD, Cremophor as solvent, and acute intraperitoneal single dose of 100 mg/kg under similar administration protocols as used elsewhere in rats and mice (Deiana et al., 2012; HloŽek et al., 2017). However, when compared to the bioavailability levels of rats and mice, the serum concentration levels of CBD following 100 mg/kg intraperitoneal injections in the GASH/Sal hamsters were very low. Our HPLC results showed that serum CBD concentrations reached peaks of 263 ng/mL (45 min post-administration), which rapidly and continuously declined until reaching values close to the detection threshold at 12 h. These results indicated both rapid absorption and elimination of CBD from blood that can be due to an accelerated metabolic degradation of the drug in the GASH/Sal. With this argument in mind, it would be worthy to carry out future experiments in the GASH/Sal using higher doses of CBD. Indeed, CBD intraperitoneal doses of 100 mg/kg or more were reported as the median effective dose of CBD for complete abolition of seizures in several rats and mice models of epilepsy (e.g., Karler et al., 1973; Kaplan et al., 2017; reviewed in Lazarini-Lopes et al., 2020). As the blood–brain barrier affects the ability of a drug to enter the brain and its integrity might be altered in epilepsy (Marchi et al., 2012), it is also critical to measure the CBD concentrations in the brain, which was a limitation of the present study. Furthermore, several studies claimed anticonvulsant effects of CBD in electrical or chemical kindling protocols that cause repetitive seizures in animals, showing a delay of kindling progression (Klein et al., 2017, Vilela et al., 2017). The genetically seizure-prone strains of rodents express different patterns of seizures, in a manner that the acute protocol precipitates generalized tonic–clonic seizures similar to grand mal epilepsy in humans, and the kindling protocol (chronic-evoked seizures) as a type of temporal lobe epilepsy model (Ross and Coleman, 2000; Garcia-Cairasco, 2002; Kandratavicius et al., 2014). In the present study, we evaluated the CBD effects on an acute protocol of seizure stimulation and using only one drug concentration, so experiments given CBD in a dose-dependent manner in GASH/Sal animals submitted to audiogenic kindling protocols might clarify the protective effectiveness of CBD against AGSs.

One of the aims of the present study was to determine the anticonvulsant effects of CBD and VPA coadministration. The fact that not all the GASH/Sal animals blocked the seizures following 300 mg/kg of VPA provided an opportunity to determine possible synergistic (additive or antagonistic) effects when administered in combination with CBD. Our results showed that the VPA and CBD coadministration did not alter the therapeutic outcome of the monotherapy with VPA, showing no apparent additive or antagonistic effects. Thus, the acute coadministered CBD and VPA drastically reduced the seizures scores, increasing the seizure latency and duration of the wild-running phase, as well as decreasing the duration of the convulsions. These effects were almost the same as those reported in GASH/Sal animals treated with VPA alone, showing the same percentage of animals in which seizures were completely eliminated. The most plausible explanation is that serum concentration levels of VPA were not modified when coadministered with CBD as it has been reported elsewhere (Gaston et al., 2017). Although controversy still remains on whether CBD potentiates the anticonvulsant effects of classic antiepileptic drugs (reviewed in Lazarini-Lopes et al., 2020), new experiments on GASH/Sal combination treatments might be useful to identify possible pharmacological interactions between CBD and conventional anticonvulsant drugs.

Common adverse effects associated with CBD treatment in epileptic patients included somnolence/sedation, decreased appetite, increases in transaminases, and diarrhea, behavioral changes, rashes, fatigue, and sleep disturbances (Devinsky et al., 2016; Franco and Perucca, 2019; Wheless et al., 2019). In our study, we reported that CBD treatments in the GASH/Sal had no effect on body weight, indicating that the administration of CBD did not affect food or water intake. Of particular concern, however, is the risk for CBD-induced hepatotoxicity, which has been reported in mice following CBD administration at high doses up to 2,460 mg/kg (Ewing et al., 2019). Our results indicated no adverse effects in liver function as well as in the hematologic profile following repeated daily administration of CBD. We further found that coadministered VPA and CBD significantly reduced hemoglobin levels and white blood cell counts. As it is known that VPA prompt neutropenia or leukopenia (Hsu et al., 2009) and the CBD-treated group showed normal values, the alterations in the hematological profile observed in the VPA+CBD-treated group were likely due to the VPA rather than CBD. Furthermore, studies in preclinical models of epilepsy reported that CBD treatment might reach full seizure protection without producing significant motor impairment (Hill et al., 2013; Klein et al., 2017). In our experiments, the open-field test revealed a decrease in the locomotor activity and exploratory behaviors after CBD administration, particularly in combination treatment with VPA. Because VPA produced motor adverse effects (Zaccara et al., 2004), the coadministration of CBD and VPA in the GASH/Sal did not seem to improve the motor impairment associated with the VPA treatment. These results are also in line with possible sedative effects of CBD on the GASH/Sal, which have been previously reported in other rodent models of epilepsy treated with CBD (Pickens, 1981; Gu et al., 2019). We also reported a significant increase in the permanence time in the center of the arena, as well as the number of rearing in the CBD- and CBD+VPA-treated animals, which may be related to increasing exploratory behavior and reduced anxiety-like behavior exerted by the drugs (Sasibhushana et al., 2019). Our results indicated modification of emotionality in GASH/Sal animals after CBD administration and its combination with VPA, but not when VPA was administered alone. Although both drugs reduce exploratory activity, the time spent in the center was considerably increased in all treated groups. This seems contradictory if we consider that more thigmotaxis and less locomotion activity are thought to indicate greater anxiety, whereas greater exploration and more ambulation in the center arena reflect less emotionality (Choleris, 2001). Other studies also reported the lack of influence of CBD on the main parameters of the open-field test such as number of square crossings, number of entries, and time spent in the center zone. Additionally, vertical exploration (i.e., rearing and wall leaning) was increased in rats by CBD (Campos and Guimarães, 2008; Shoval et al., 2016; Sales et al., 2018). It is worth mentioning that the differences between the open-field performance that were reported here in the GASH/Sal hamster and those reported in other studies (mainly rats and mice) might be related to interspecies differences.

An important outcome of the present study was the mRNA expression analysis of genes in the inferior colliculus of the GASH/Sal after each chronic treatment. The inferior colliculus is considered the brain area for seizure generation and propagation in rodent models of AGS susceptibility (reviewed in Garcia-Cairasco, 2002). Recent studies in the GASH/Sal pointed out that the inferior colliculus is embedded in a web of pathologic connections in the auditory pathway that constitutes a seizure-prone network to finally drive the AGS (Sánchez-Benito et al., 2020). In an attempt to shed light on the molecular effects of each treatment in the inferior colliculus of the GASH/Sal, we analyzed the gene expression levels of *Trpv1, 5-Htr1a, Sigmar1, Adora1, Slc29a1*, and *Cnr1*. Although there is a very long list of neuronal receptors that are targets of CBD or are related to its action mechanism (reviewed in Franco and Perucca, 2019), we selected those genes because they encoded proteins related to regulation of neuronal excitability and neuroprotection that might be presumably affected by the pharmacological activity of the treatments on the epileptogenic focus. TRPV1 is a non-selective channel with high permeability to Na^+^ and Ca^2+^, and its activation promotes the release of glutamate by increasing the neuronal excitability (Caterina et al., 1997; Naziroglu et al., 2013). CBD exerts effects on TRPV1 generating receptor activation and desensitization (Iannotti et al., 2014), and hence binding at TRPV1 might regulate neuronal activity. Our PCR results showed significant increase in mRNA expression of *Trpv1* gene following the coadministration of CBD and VPA, suggesting a joint action of both drugs that might lead to increased neuronal susceptibility and neurotoxicity (Naziroglu and Övey, 2015). In fact, these results correlated to our behavioral results showing that chronic coadministration of CBD and VPA had subtle or no effects on the AGS of the GASH/Sal. In line with this argument, Sun et al. (2013) reported higher gene expression levels of *Trpv1* in the hippocampus and the cortex of drug-resistant mesial temporal lobe epilepsy patients. Activation of the serotonin 5-HTR1A receptor hyperpolarizes the resting membrane potential and has an anticonvulsant effect in various experimental *in vivo* and *in vitro* seizure models (Salgado and Alkadhi, 1995; Gariboldi et al., 1996). Although CBD has been shown to be an agonist of the 5-HTR1A receptor (Russo et al., 2005), studies in the pentylenetetrazole model of generalized seizures reported that 5-HTR1A is not involved in CBD's anticonvulsant effect (Pelz et al., 2017). Consistently with this, our results showed normal mRNA expression levels of 5-HTR1A after chronic CBD treatment. SIGMAR1 receptor antagonists inhibit glutamate *N*-methyl-d-aspartate acid receptor (NMDAR) activity and display positive effects on epilepsy. Although CBD displays antagonist-like activity toward SIGMAR1 receptor to reduce the negative effects of NMDAR overactivity in epilepsy (Rodríguez-Muñoz et al., 2018), our results showed that chronic CBD administration or coadministration with VPA did not affect the gene expression levels of SIGMAR1. The ADORA1 receptors are involved on the adenosine modulation system and contribute to adaptive changes in neurotransmission and neuroprotection. Thus, adenosine acts as a negative regulator of glutamate release via activation of the presynaptic ADORA1 receptors. Histopathologic and biochemical analyses of surgical resections of patients with epilepsy showed a decrease in the gene expression of ADORA1, suggesting that this decrease contributes to chronic epilepsy in humans (Glass et al., 1996; Boison, 2016). CBD bolsters adenosine signaling by inhibiting its extracellular removal and provides an ADORA1 receptor–mediated mechanism by which CBD decreases inflammation (Liou et al., 2008). In this context, the high levels of *Adora1* transcripts in the GASH/Sal inferior colliculus after chronic CBD administration might be a compensatory mechanism for overactivation of glutamate receptors and/or the increment of glutamate release. Adenosine shows anticonvulsant effects, and it is released during seizures (Ilie et al., 2012). Furthermore, Carrier et al. (2006) revealed that CBD binds to the equilibrative nucleoside transporter 1 (encoded by *Slc29a1* gene), which led to the increase of extracellular adenosine. Thus, our results showing a decrease in gene expression levels of *Slc29a1* in GASH/Sal animals treated with CBD alone or the combination of CBD and VPA might be a mechanism to regulate neuronal excitability in the epileptogenic nucleus. The *Cnr1* gene encoded the central cannabinoid receptor CB1, which is activated by anandamide and inhibits the presynaptic release of glutamate and γ-aminobutyric acid (Howlett and Abood, 2017). In animal models of epilepsy, the gene expressions of *Cnr1* and *Trpv1* are increased (von Rüden et al., 2015). Furthermore, CBD behaves as a non-competitive negative allosteric modulator of CB1 receptors (Laprairie et al., 2015) and inhibits reuptake and hydrolysis of anandamide (Bisogno et al., 2009). Thus, our PCR results showing that CBD treatment causes an increase of *Cnr1* gene expression might be related to a possible protective effect against increased neuronal excitation and an attempt to inhibit glutamate release in the GASH/Sal inferior colliculus.

Together, our results are in line with previous studies conducted on epileptic seizure models showing that the CBD's mechanism of action is complex, as its anticonvulsant activity is mediated by multifactorial mechanisms underlying the behavioral, electrophysiological, and neuroprotective effects of CBD (reviewed in Lazarini-Lopes et al., 2020). New experiments at higher dosages as well as in the audiogenic kindling protocol (chronic-evoked seizures) might be necessary to delve deeper into the anticonvulsant effects of CBD in the GASH/Sal model.

## Data Availability Statement

The original contributions presented in the study are included in the article/Supplementary Materials, further inquiries can be directed to the corresponding author/s.

## Ethics Statement

The animal study was reviewed and approved by Bioethics Committee of the University of Salamanca (application number 380).

## Author Contributions

DL, RG-N, and CS conceived the original idea, designed the experiments, and supervised the project. DL and RG-N secured funding. RG-N wrote the manuscript and performed visualization/data presentation. GC-P and DS-B carried out the drug administration experiments and analyzed the seizure severity index. GC-P carried out blood-sampling procedure and the formal analysis of the hematological and biochemical profiles. SD-R performed the RT-qPCR experiments. LM provided the animals and performed the bibliographic review. OC and GC-P carried out the open-field test and the formal analysis of the open-field results. CS designed and supervised the pharmacological part of the project. JG evaluated the seizure behaviors with the Ethomatic and performed the ethograms. DL, RG-N, CS, OC, and LM provided critical feedback and helped shape the manuscript. All authors reviewed and approved the paper.

## Conflict of Interest

DS-B was hired under the research funding from RiverForce Partners Inc. The remaining authors declare that the research was conducted in the absence of any commercial or financial relationships that could be construed as a potential conflict of interest.

## Supplementary Material

Supplementary Material 1 Video recording of a representative audiogenic seizure in a GASH/Sal animal. Following acute sound stimulation the GASH/Sal animal showed five differenced and consecutive phases: (1) a behavioral arrest, (2) a period of wild running, (3) tonic–clonic convulsions, (4) head ventral flexion, forelimb extension, and hind limb extension, and (5) postictal immobility. The GASH/Sal animal shown in the video did not receive drug treatment and belonged to the baseline (pre-treatment) condition. Notice that the behavioral items corresponding to each seizure phase are simultaneously depicted in the neuroethological analysis (flowchart on top right corner of the video). See Figure 2 for detailed interpretation of the flowchart.

Supplementary Material 2 Table shows the percentage of animals based on the categorized seizure scores > 2 and <2 in each of the experimental groups.

Supplementary Material 3 Raw data of RT-qPCR used for analyses. The qPCR data included a set of 4 to 6 biological replicates (sample cases) for each experimental group (treatment condition), triplicate technical replicates as well as the Ct values of housekeeping gene beta-actin used for data normalization. *Adora1*, the adenosine A1 receptor; *Actb*, β-actin; CBD, cannabidiol; *Cnr1*, the cannabinoid receptor 1; *Sigmar1*, the sigma non-opioid intracellular receptor 1; *Slc29a*, the equilibrative nucleoside transporter 1; *Trpv1*, the transient receptor potential of vanilloid type 1; VPA, valproic acid; 5-HTR1A, the 5-hydroxytryptamine (serotonin) receptor 1A.
