# Supplementary figures and images for: Behavioral and Molecular Effects Induced by Cannabidiol and Valproate Administration in the GASH/Sal Model of Acute Audiogenic Seizures
Source: Front Behav Neurosci. 2021 Jan 22;14:612624. doi: 10.3389/fnbeh.2020.612624 (PMC7862126; doi:10.3389/fnbeh.2020.612624)

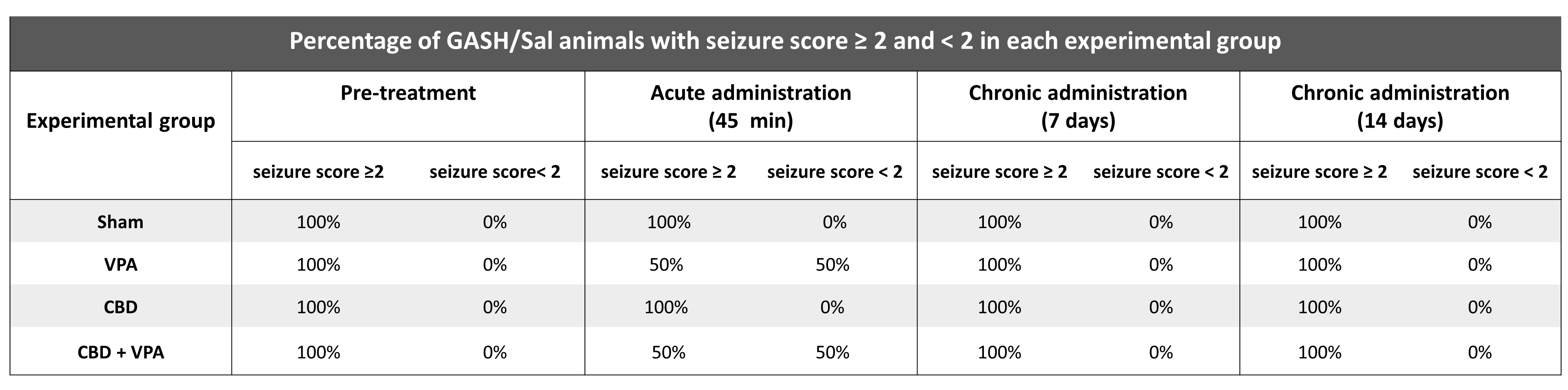

Supplement: Supplementary Material 2 — Table shows the percentage of animals based on the categorized seizure scores > 2 and <2 in each of the experimental groups. [file Image_1.TIF]
