# Supplementary material for: Behavioral and Molecular Effects Induced by Cannabidiol and Valproate Administration in the GASH/Sal Model of Acute Audiogenic Seizures
Source: Front Behav Neurosci. 2021 Jan 22;14:612624. doi: 10.3389/fnbeh.2020.612624 (PMC7862126; doi:10.3389/fnbeh.2020.612624)
Supplement: Supplementary Material 3 — Raw data of RT-qPCR used for analyses. The qPCR data included a set of 4 to 6 biological replicates (sample cases) for each experimental group (treatment condition), triplicate technical replicates as well as the Ct values of housekeeping gene beta-actin used for data normalization. Adora1, the adenosine A1 receptor; Actb, β-actin; CBD, cannabidiol; Cnr1, the cannabinoid receptor 1; Sigmar1, the sigma non-opioid intracellular receptor 1; Slc29a, the equilibrative nucleoside transporter 1; Trpv1, the transient receptor potential of vanilloid type 1; VPA, valproic acid; 5-HTR1A, the 5-hydroxytryptamine (serotonin) receptor 1A. [file Data_Sheet_1.PDF]

Raw data of RT-qPCR used for analyses (Ct values)

Animal group (treatment)	Animal ID	Replicate	*Trpv1*	*5-Htr1a*	*Sigmar1*	*Adora1*	*Slc29a1*	*Cnr1*	*Actb*
**Sham (vehicle) ***	4359	1	25,132	22,331	18,959	21,463	22,491	22,904	14,889
		2	25,311	22,186	18,945	21,916	22,673	23,010	15,091
		3	25,491	22,290	18,929	22,361	22,861	23,105	15,280
	4362	1	25,676	Undetermined*	19,320	21,442	24,093	23,764	16,834
		2	25,776	Undetermined*	19,415	21,598	24,572	23,756	16,751
		3	25,876	Undetermined*	19,403	21,753	25,051	23,734	16,657
	4373	1	25,578	23,132	19,269	22,399	23,244	22,996	15,949
		2	25,366	23,064	19,310	22,292	23,349	23,143	15,742
		3	25,154	22,997	19,345	22,185	23,439	23,283	15,523
	4375	1	25,576	22,400	18,894	20,981	22,770	23,262	15,522
		2	25,383	22,562	19,020	20,809	22,776	23,149	15,212
		3	25,191	22,724	19,133	20,626	22,784	23,003	15,190
	4376	1	27,038	23,355	19,475	21,999	23,396	25,449	16,443
		2	27,085	23,362	19,199	22,068	23,359	24,987	16,311
		3	27,133	23,370	18,939	22,136	23,340	25,012	16,172
**Single CBD treatment *<br>(100 mg/kg every 12 h)**	4372	1	25,010	23,037	19,018	21,452	27,534	22,926	15,940
		2	25,870	23,293	18,926	21,276	27,295	22,965	16,420
		3	25,620	23,549	18,842	21,101	27,049	22,960	16,209
	4378	1	26,140	24,965	21,688	19,675	23,986	23,494	16,219
		2	25,883	24,370	20,375	19,345	23,959	22,869	16,121
		3	25,627	24,460	21,219	19,606	23,928	22,246	16,023
	4386	1	26,070	23,376	18,993	19,327	23,762	22,263	15,938
		2	25,811	23,128	19,065	19,996	23,880	22,398	15,784
		3	25,552	22,881	19,134	19,605	23,915	22,519	15,630
	4381	1	25,005	24,132	20,006	19,584	24,375	24,198	16,307
		2	25,008	24,203	20,045	20,248	25,560	24,459	16,569
		3	25,006	24,275	20,089	20,910	26,745	24,707	16,821
**Single VPA treatment<br>(300 mg/kg every 24 h)**	4360	1	25,938	23,038	19,474	22,511	24,372	Undetermined*	16,256
		2	25,953	23,065	19,259	22,534	24,078	Undetermined*	16,323
		3	25,951	23,093	19,047	22,536	23,763	Undetermined*	16,390
	4363	1	24,626	Undetermined*	19,021	Undetermined*	24,645	23,038	15,111
		2	24,485	Undetermined*	18,669	Undetermined*	23,020	22,612	15,138
		3	24,345	Undetermined*	18,302	Undetermined*	21,394	22,168	15,168
	4364	1	25,768	23,102	19,385	21,262	23,100	23,223	16,007
		2	25,783	23,198	19,093	21,149	23,170	23,389	16,059
		3	25,799	23,295	18,800	21,052	23,230	23,568	16,098
	4374	1	25,839	23,329	19,168	21,642	23,466	22,081	15,772
		2	25,185	23,151	19,431	21,439	23,289	22,221	15,845
		3	25,532	23,273	19,695	21,241	23,133	22,356	15,901
	4387	1	25,112	22,992	18,027	21,280	22,739	24,557	15,471
		2	25,172	22,940	18,186	21,018	22,896	24,228	15,570
		3	25,233	22,889	18,336	20,756	23,042	24,238	15,669
	4379	1	25,696	Undetermined*	19,513	Undetermined*	23,467	23,079	16,347
		2	25,706	Undetermined*	19,325	Undetermined*	23,748	23,512	16,301
		3	25,717	Undetermined*	19,114	Undetermined*	23,999	23,937	16,262
**CBD + VPA combined treatments *<br>(CBD: 100 mg/kg every 12 h)<br>(VPA: 300 mg/kg every 24 h)**	4371	1	26,039	26,298	22,790	26,078	28,233	26,039	18,573
		2	25,821	25,942	22,766	25,783	27,789	25,879	18,429
		3	25,604	25,587	22,681	25,488	27,347	25,713	18,264
	4383	1	23,911	24,298	19,276	24,128	25,097	24,369	16,569
		2	23,923	24,112	19,206	24,109	24,795	23,896	16,518
		3	23,835	23,926	19,135	24,076	24,492	23,730	16,452
	4384	1	25,527	26,337	20,721	Undetermined*	26,993	25,731	18,605
		2	25,587	26,475	20,701	Undetermined*	27,093	25,823	18,498
		3	25,648	27,100	20,681	Undetermined*	27,193	25,806	18,372
	4380	1	Undetermined*	26,398	22,621	26,803	28,668	Undetermined*	19,265
		2	Undetermined*	26,192	22,746	26,113	28,211	Undetermined*	19,439
		3	Undetermined*	26,287	22,863	25,424	28,890	Undetermined*	19,399
	4382	1	27,132	Undetermined*	23,481	27,580	29,108	27,456	19,654
		2	27,205	Undetermined*	23,419	27,439	29,849	27,587	19,658
		3	27,278	Undetermined*	23,353	27,315	29,595	27,725	19,642

* Outlier and undetermined values (below the detection limit of the qPCR assay) of biological replicates were not included in the data sheet.
